# A Study of the Effects of Hf and Sn on the Microstructure, Hardness and Oxidation of Nb-18Si Silicide Based Alloys without Ti Addition

**DOI:** 10.3390/ma11122447

**Published:** 2018-12-03

**Authors:** Eleftherios Zacharis, Claire Utton, Panos Tsakiropoulos

**Affiliations:** Department of Materials Science and Engineering, Sir Robert Hadfield Building, University of Sheffield, Mappin Street, Sheffield S1 3JD, UK; lefteris.zacharis@alfagro.gr (E.Z.); c.utton@sheffield.ac.uk (C.U.)

**Keywords:** high temperature alloys, silicides, intermetallics, Nb silicide in-situ composites, pest oxidation, hardness

## Abstract

The paper presents the results of an experimental study of large (≈0.6 kg) arc melted buttons of four Ti free Nb-silicide based alloys with Sn addition with nominal compositions (at.%) Nb-18Si-5Hf-5Sn (EZ1), Nb-18Si-5Al-5Sn (EZ7), Nb-18Si-5Cr-5Hf-5Sn (EZ3) and Nb-18Si-5Al-5Hf-5Sn (EZ4). The alloys were studied in the as-cast and heat treated conditions. In all the alloys there was macrosegregation of Si (MACSi). Among the single element additions Hf had the weakest and Sn the strongest effect on MACSi. The simultaneous presence of Cr and Hf in the alloy EZ3 had the strongest effect on MACSi. In all the alloys the βNb_5_Si_3_ was the primary phase and was present after the heat treatment(s), the Nb_3_Si silicide was suppressed and the A15-Nb_3_Sn intermetallic was stable. The Nb_ss_ was not stable in the alloys EZ7 and EZ4 and the C14-NbCr_2_ Laves phase was stable in the alloy EZ3. Very Hf-rich Nb_5_Si_3_ was stable in the alloy EZ4 after prolonged heat treatments. Eutectics were observed in all the alloys. These were binary eutectics in the alloys EZ1 and EZ7, where respectively they consisted of the Nb_ss_ and βNb_5_Si_3_, and βNb_5_Si_3_ and A15-Nb_3_Sn phases. Most likely ternary eutectics consisting of the Nb_ss_, C14-NbCr_2_ and βNb_5_Si_3_, and Nb_ss_, βNb_5_Si_3_ and A15-Nb_3_Sn phases were observed, respectively in the alloys EZ3 and EZ4. The addition of Al increased the vol% of the Nb_5_Si_3_ and A15-Nb_3_Sn phases, particularly after the heat treatment(s). The lattice parameter of Nb respectively increased and decreased with the addition of Hf, and Al or Cr and the latter element had the stronger negative effect. Pest oxidation was not suppressed in the alloys of this study.

## 1. Introduction

Niobium silicide based alloys (or Nb-silicide in situ composites) currently are developed owing to their potential to replace Ni-based superalloys in future aero-engines to enable the latter to meet new environmental and performance targets. These new ultra-high temperature alloys must meet property goals for toughness, creep resistance and oxidation. The property goals were given in [1]. The aforementioned properties depend on the chemical composition, distribution (size and spatial) and volume fraction of the phases that are present in the microstructures of the alloys, where the most desirable ones are the bcc Nb solid solution (Nb_ss_) for toughness, and the tetragonal Nb_5_Si_3_ silicide for creep resistance and oxidation. A high volume fraction of the Nb_ss_ has negative effect on the creep resistance and oxidation of the alloys. The volume fraction of Nb_5_Si_3_ is crucial for the toughness and creep of the alloys.

The progress made on the development of Nb-silicide based alloys until the start of the 21st century was reviewed by Bewlay and Jackson [1]. More recently the alloying behaviour of Nb-silicide based alloys and their key phases was studied in [2,3,4,5,6] where links between alloying and properties were discussed and the usefulness of these studies for the design and/or selection of new alloys was demonstrated in [6]. Also, it was shown that some of the studied Nb-silicide based alloys, and some of the bcc Nb solid solutions and Nb_ss_ + βNb_5_Si_3_ eutectics that are formed in Nb-silicide based alloys [7] satisfy the standard definition of the so-called High Entropy Alloys (HEAs) [6].

Nb-silicide based alloys can offer a balance of properties and at the same time satisfy some of the property goals. Hafnium, Sn and Ti are important additions in these alloys for achieving a balance of properties. Each of these three elements improves oxidation, particularly in the presence of Al and/or Cr [8,9,10,11]. Ti in synergy with Al and/or Cr cannot suppress pest oxidation [11] but Sn can when it is in synergy with these elements [9]. The concentrations of Hf and Ti are important for creep resistance [6,12]. Also, primary phase selection and phase stability in the alloys depends on the synergies of the aforementioned elements.

To date, most of the research on Nb-silicide based alloys with Hf and/or Sn additions is on Ti containing alloys. Ti and Hf behave similarly in these alloys. For example, both elements can stabilize the hexagonal γNb_5_Si_3_, their concentrations and those of Al and/or Cr in the Nb_ss_ are inter-dependent [6] and the partitioning of Ti and Hf in the microstructure can make the identification of phases very difficult, particularly when Sn is also present [11,13]. There is some limited research in Ti-free Nb-silicide based alloy where (i) Hf was in synergy (a) only with Si or (b) with Al and Si or (c) with Cr and Si and (ii) Sn was in synergy only with Si. Indeed, the Ti-free ternary alloys of nominal compositions Nb-18Si-5Sn (alloy NV9 in [14]), Nb-19Si-5Hf and Nb-16Si-xHf (x = 1, 3, 7) [15,16] and the Ti-free quaternary alloys of nominal compositions Nb-18Si-5Cr-5Hf and Nb-18Si-5Al-5Hf (respectively the alloys YG1 and YG2 in [17]) have been studied (in this paper all compositions are given in at.% unless otherwise stated).

In Nb-19Si the primary phase is Nb_3_Si [18]. The calculated solidification path for the alloy Nb-19Si-5Hf indicated the Nb(Hf)_3_Si silicide as the primary phase [15]. Increase of the Hf concentration in Nb-16Si-xHf (x =1, 3, 7) alloys refined the microstructure, decreased the volume fraction of the Nb_ss_ + Nb_3_Si eutectic and improved the fracture toughness of the alloys. The latter was attributed to the Hf addition promoting a transition of the Nb_ss_ fracture from brittle cleavage to plastic stretching [16]. When Al was added to the Nb-18Si-5Al-5Hf alloy (alloy YG2 in [17]), the Nb_3_Si was suppressed, the primary phase was the βNb_5_Si_3_ and the volume fraction of the Nb_ss_ was reduced. However, when Cr replaced Al in the Nb-18Si-5Cr-5Hf alloy (alloy YG1 in [17]) the primary phase was the βNb_5_Si_3_ but the Nb_3_Si formed in parts of the button that had not solidified under high cooling rate. The Nb_3_Si was not stable in the heat treated alloy YG1. 

With the addition of Sn in the alloy Nb-18Si-5Sn the primary phase was the βNb_5_Si_3_ and the Nb_3_Si was suppressed, the Nb_ss_ + Nb_3_Si eutectic was replaced by the Nb_ss_ + βNb_5_Si_3_ eutectic and the A15-Nb_3_Sn phase was also stable in the microstructure [14] (see discussion and the Supplemental data). Tin partitioned to the bcc Nb_ss_ stronger than to the Nb_5_Si_3_ and did not significantly affect the solubility of Si in the Nb_ss_ [14]. In other words, the research has shown (i) that the suppression of the Nb_3_Si was promoted by the synergy (a) of Sn with Si, (b) of Al with Hf and Si and (c) of Hf with Si but not when Cr and Hf were in synergy with Si and (ii) that the primary phase was the βNb_5_Si_3_ in Nb-silicide based alloys where all the aforementioned elements (i.e., Al, Cr, Hf, Si, Ti) were in synergy. It should be noted that the alloys NV9, YG1 and YG2 were prepared as arc melted buttons of approximately 0.6 kg weight (see discussion). Recently it was shown that the βNb_5_Si_3_ to αNb_5_Si_3_ transformation in the alloys depends on the size of arc melted button [19].

How would the simultaneous presence of Hf and Sn with/out Al or Cr (i.e., of the elements that are key to improving the oxidation of Nb-silicide based alloys) affect the microstructure and properties of Ti-free Nb-silicide based alloys? Would the macrosegregation of Si increase or decrease? Would eutectics form in such alloys? Which would be the phases in the eutectics? Would the Nb_ss_ be stable in the alloys? Would the stability of βNb_5_Si_3_ be increased or decreased? What would be the effect on hardness? Would pest oxidation be suppressed? The motivation for the research presented in this paper was (i) to answer these questions, (ii) to show the strong effect of the partitioning of Hf and Sn in the microstructure in the absence of Ti and (iii) to highlight the implication of (ii) for phase identification.

The structure of the paper is as follows. First, the microstructures of the four studied alloys in the as cast and heat treated conditions are presented and then the results for their hardness, densities and isothermal oxidation at 800 °C. Macrosegregation and the cast and heat treated microstructures are discussed followed by the hardness and oxidation of the alloys. We have decided to present the results for each alloy separately because we used different heat treatment temperatures and times for different alloys to study the stability of the Nb solid solution and the type of Nb_5_Si_3_ in their microstructures and also in order to address (ii) and (iii) above. 

## 2. Experimental

The alloys of nominal compositions Nb-18Si-5Hf-5Sn (alloy EZ1), Nb-18Si-5Al-5Sn (alloy EZ7), Nb-18Si-5Cr-5Hf-5Sn (alloy EZ3) and Nb-18Si-5Al-5Hf-5Sn (alloy EZ4) were selected for this study. The effect of Hf or Al on microstructure and properties was studied using the alloys EZ1 and EZ7 that were compared with the Nb-18Si-5Sn alloy (alloy NV9 in [14]). The simultaneous addition of Al and Hf reduced significantly the volume fraction of the Nb_ss_ in the alloy Nb-18Si-5Al-5Hf compared with the addition of Cr and Hf in the alloy Nb-18Si-5Cr-5Hf (respectively the alloys YG2 and YG1 in [17]). The alloy EZ7 was selected to find out how Al and Sn affect the stability of the Nb_ss_ when present simultaneously in the alloy without Hf. The effect of Sn with Cr and Hf, or Al and Hf on microstructure and properties was studied in the alloys EZ3 and EZ4.

Large buttons of the aforementioned alloys of approximately 0.6 kg weight were prepared from elemental charges of purity better than 99.99 wt.% using arc melting with a non-consumable tungsten electrode and water cooled copper crucible in an argon atmosphere. Cubic specimens from the bulk of the ingot of each alloy were used for heat treatments at 1200 or 1500 °C and up to 300 h depending on alloy and for isothermal oxidation experiments. For the heat treatments cubic (2 × 2 × 2 cm^3^) specimens wrapped in Ta foil were placed in a LENTON 1850 high temperature tube furnace under a constant flow of Ti gettered argon (10^−5^ m^3^·s^−1^). The oxidation experiments were done at 800 °C in static air for up to 100 h using cubic (3 × 3 × 3 mm^3^) specimens of the heat treated alloys in a Stanton-Redcroft automatic thermo-recording balance.

The microstructures were characterized using X-ray diffraction (XRD) and electron probe micro-analysis (EPMA). For the XRD a Siemens D5000 diffractometer with Cu radiation was used and X-rays were collected with a step of 0.02 degrees over 2θ range 20 to 90 degrees. Peaks in the XRD diffractograms were identified by correlating data from the experiments with that from the JCPDS data (International centre for diffraction data). The lattice parameter of the Nb_ss_ was determined using the Nelson-Riley function [20]. Secondary electron (SE) and backscatter electron (BSE) imaging and quantitative analysis were undertaken using a JEOL 8600 EPMA equipped with energy-dispersive (EDS) and wavelength-dispersive (WDS) spectrometers. Standards of high purity elements of Nb, Si, Cr, Al, Hf and Sn, which had been polished to a finish of 1 μm, were used. The operational software was the Oxford Link INCA software with the XPP corrections method which is based on the Rhi-Rho-Z approach. At least 10 analyses for each phase or area of the ingot were performed. The chemical analysis data is given in the Tables S1–S4 in the Supplemental data. In these Tables the data is for the phases in the whole of each cast button and the average value, standard deviation and the minimum and maximum analyses values are given. The data in the Tables S1–S4 is for the phases that were identified both by XRD and EPMA.

The Vickers hardness (HV) of all the alloys in the as cast and the heat treated conditions was measured using a CV-430 AAT automatic hardness testing machine. The load used was 10 kg and was applied for 20 s. At least 10 measurements were taken for each alloy. The hardness of phases in the alloys were measured using a Mitutoyo micro-hardness testing machine. The load used was 0.1 kg and was applied for 20 s. At least 10 measurements were taken for each phase.

A Sartorius Master^pro^ Series electronic analytical balance along with a Sartorius YDK density determination kit was used to calculate the density of the alloys. The Archimedean principle was applied for measuring the density of the alloys.

## 3. Results

The actual compositions of the alloys EZ1, EZ7, EZ3 and EZ4 respectively were 70Nb-20.5Si-5.4Hf-4.1Sn, 72.1Nb-18.9Si-5Al-4Sn, 66.2Nb-19.7Si-4.5Cr-5.3Hf-4.5Sn and 67.6Nb-19.4Si-4.4Al-5.3Hf-3.3Sn (see Tables S1–S4 in the Supplemental data). Compared with their nominal compositions, the cast alloys were poorer in Sn and richer in Si. There was macrosegregation of Si (MACSi = C_Si_^max^ − C_Si_^min^, where C_Si_^max^ and C_Si_^min^ respectively are the maximum and minimum measured concentrations in the cast button [21]) in all alloys and macrosegregation of Cr in the alloy EZ3. The values of MACSi were 2.8, 2.5, 4.1 and 3.9 at.% respectively for the alloys EZ1, EZ7, EZ3 and EZ4. The phases in the microstructures of the alloys are summarized in the Table 1. The Nb_3_Sn and Nb_5_Si_3_ were stable in all alloys.

**Figure 1:**
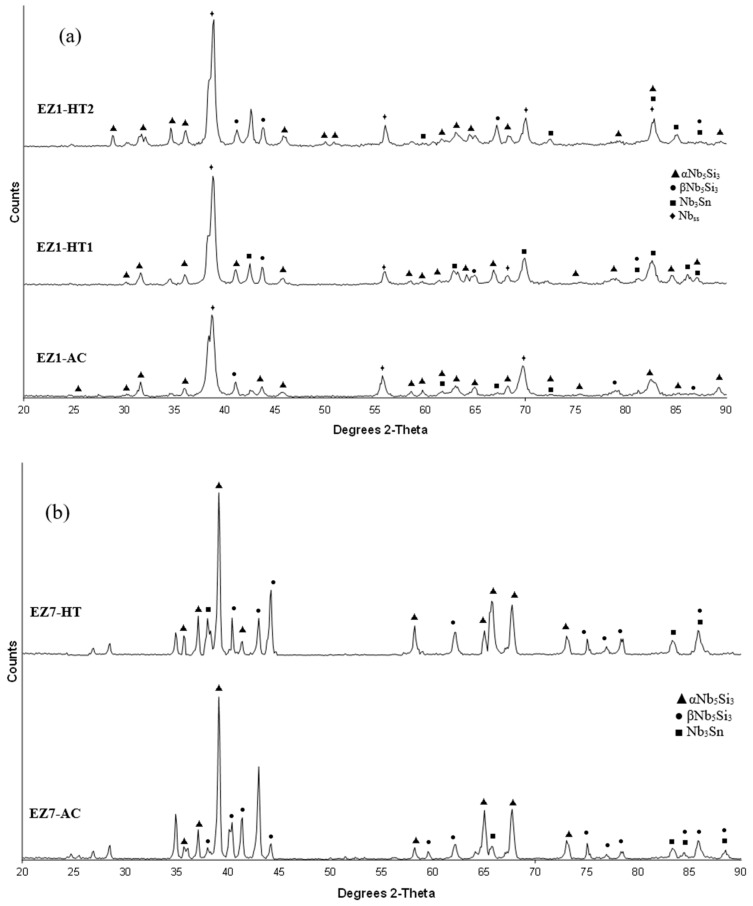
X-ray diffractograms of the as-cast and heat-treated alloys (**a**) EZ1 and (**b**) EZ7.

**Figure 2:**
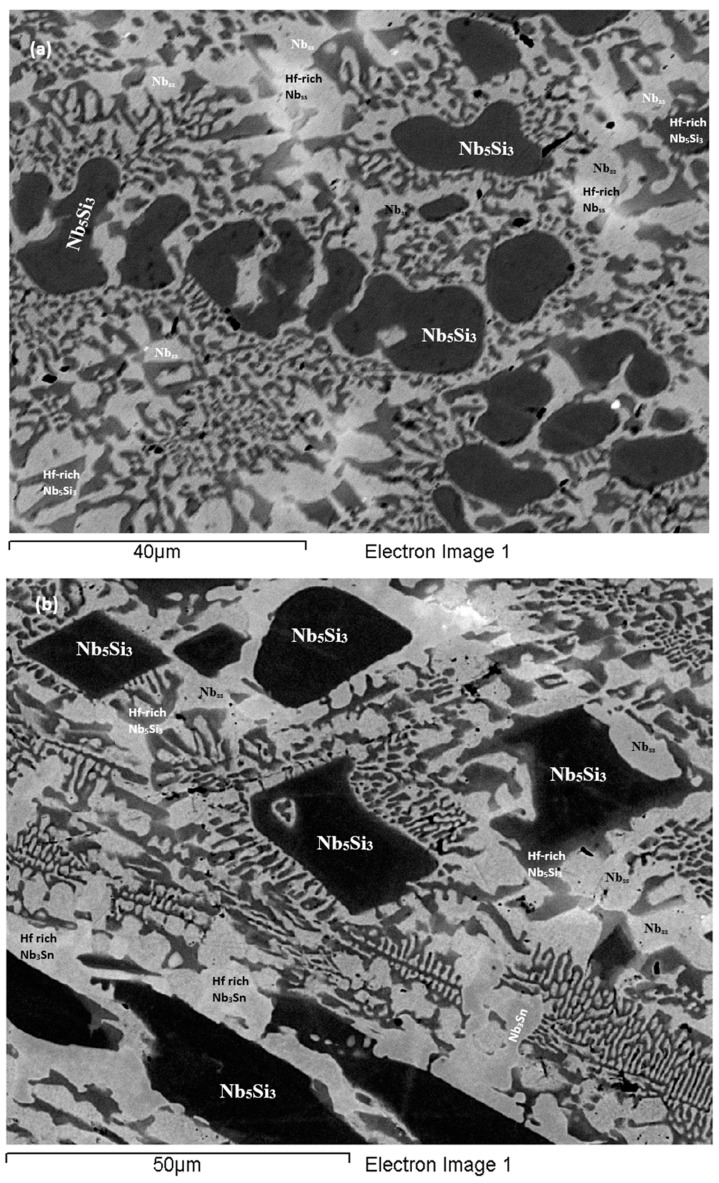

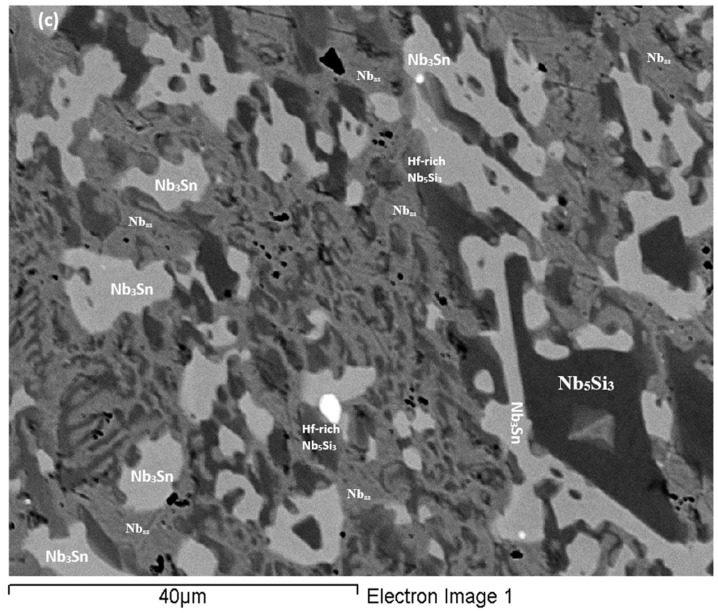
BSE images of the microstructure of the top (**a**) and bulk (**b**) of the as-cast and (**c**) bulk of the heat treated (1500 °C/100 h) alloy EZ1.

**As-cast alloy EZ1 (EZ1-AC):** According to the XRD data the phases in the cast microstructure were the Nb_ss_, αNb_5_Si_3_, βNb_5_Si_3_, Nb_3_Sn and HfO_2_ (Figure 1a). More peaks corresponded only to αNb_5_Si_3_ than only to βNb_5_Si_3_. The cast microstructure is shown in Figure 2a,b. The Nb_ss_ and Nb_3_Sn exhibited essentially the same contrast under BSE imaging, meaning these phases could be distinguished only by performing chemical analysis. The Si concentration in the Nb_ss_ was in agreement with data for Nb-silicide based alloys (e.g., [11,17]). The presence of Nb_3_Sn was confirmed by EPMA analyses only in the bulk of the ingot (Figure 2b). There were Hf-rich areas in the Nb_ss_, Nb_5_Si_3_ and Nb_3_Sn. The Sn concentration in these Hf rich phases increased with their Hf concentration, and in the Hf rich Nb_3_Sn the Si concentration was reduced. The Hf rich areas exhibited a brighter contrast under BSE imaging (Figure 2a,b). The volume fraction of the Nb_5_Si_3_ was higher than those of the Nb_3_Sn and Nb_ss_, which were the same (see Table A1 in the Appendix A).

A fine lamellar microstructure the compositions of which were essentially the same in the top, bulk and bottom of the button surrounded the Nb_5_Si_3_ (Figure 2a,b). In the bulk it was not possible to confirm whether the lamellar microstructure was binary consisting of the Nb_ss_ and Nb_5_Si_3_ phases, as it was in the top and bottom of the button, or ternary consisting of the Nb_ss_, Nb_5_Si_3_ and Nb_3_Sn phases. Also, in the lamellar microstructure it was not possible to confirm whether some or all of the phases in it were Hf-rich because of the scale of the lamellae and the contrast of phases. The reason why in Table S1 in the Supplemental data the analysis of the lamellar microstructure in EZ1-AC is given for eutectic with Nb_ss_ and Nb_5_Si_3_ will become clear in the discussion.

**Heat-Treated alloy EZ1-HT1 (1500 °C/100 h):** The XRD data indicated that the microstructure consisted of Nb_ss_, Nb_3_Sn, αNb_5_Si_3_ and βNb_5_Si_3_ and HfO_2_ (Figure 1a). There was still chemical inhomogeneity of Si, the concentration of which varied between 19.8 and 24 at.%, and Hf rich areas in Nb_5_Si_3_ (Table S1 in the Supplemental data). The average composition of HfO_2_ was 1.8Nb-2.5Si-32.5Hf-63.2O (at.%). The microstructure had coarsened and the lamellar microstructure that was evident throughout the cast button had not disappeared completely (Figure 2c). The average composition of the remnants of the lamellar microstructure was richer in Hf and Si and poorer in Sn (Table S1 in the Supplemental data).

**Heat-Treated alloy EZ1-HT2 (1500 °C/200 h):** The alloy was given a second heat treatment for an additional 100 h in order to further homogenize its microstructure and find out whether the βNb_5_Si_3_ and the Nb_ss_ would be stable. The EPMA data for EZ1-HT2 is given in Table S1 in the Supplemental data and the XRD data in Figure 1a. The chemical inhomogeneity of Si was reduced compared with EZ1-HT1. The microstructure was similar to that in EZ1-HT1, and consisted of the same phases (Figure 1a) with Hf rich areas still present in Nb_5_Si_3_. The XRD indicated that the βNb_5_Si_3_ silicide and the Nb_ss_ were present. There were no significant changes in the composition of the phases, with the exception of the Si concentration in the Nb_ss_ that was further reduced. Areas of prior eutectic microstructure were still present, but with reduced Sn and increased Si concentration compared with EZ1-AC. Thus, the lamellar microstructure that was observed in the cast alloy EZ1 was thermally stable after 200 h at 1500 °C. After this heat treatment, the volume fraction of the Nb_5_Si_3_ was half that in the cast alloy and the volume fraction of Nb_3_Sn was reduced slightly (see Table A1 in the Appendix A). The volume fraction of the prior eutectic microstructure was about 0.58, the same as in EZ1-HT1.

**Figure 3:**
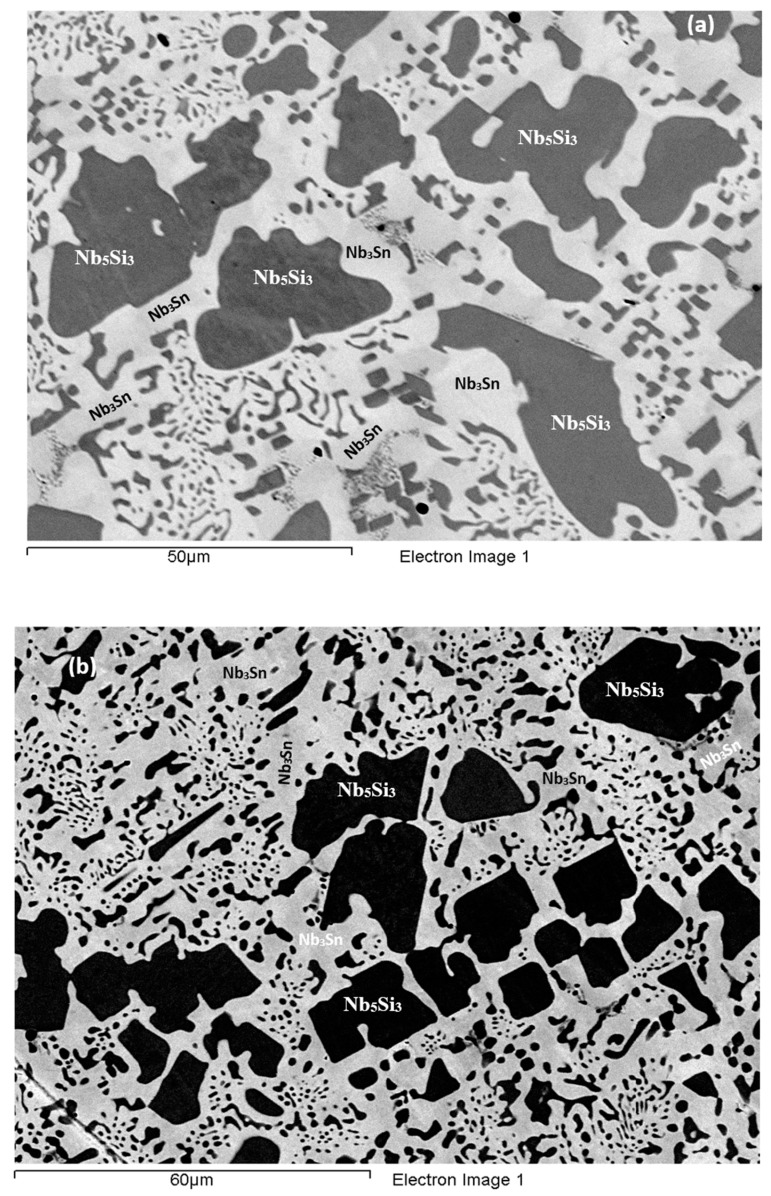

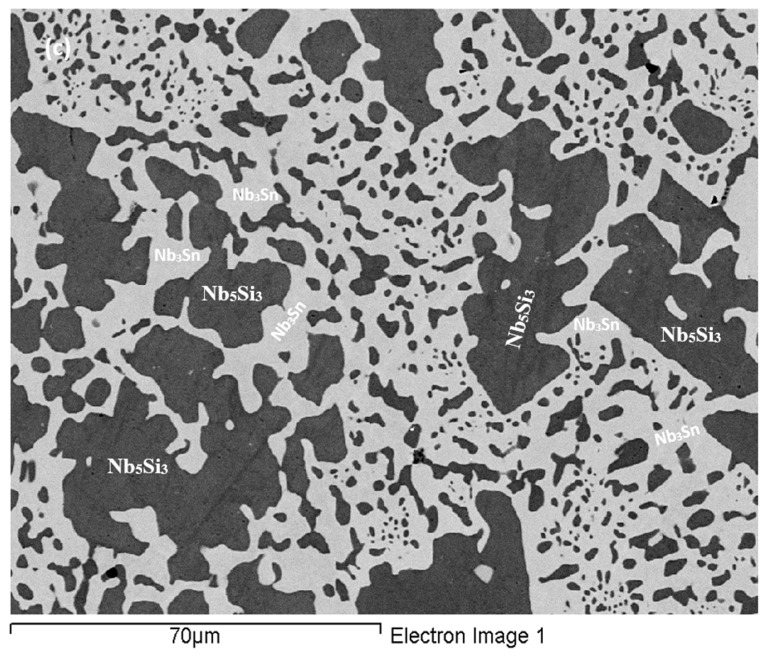
BSE images of the microstructure of the top (**a**) and bulk (**b**) of the cast and (**c**) bulk of the heat treated (1500 °C/100 h) alloy EZ7.

**As-cast alloy EZ7 (EZ7-AC):** Typical microstructures in different areas of the button are shown in Figure 3a,b and the phases are summarized in Table 1. The phases were the αNb_5_Si_3_, βNb_5_Si_3_ and Nb_3_Sn (Figure 1b). Approximately the same number of peaks corresponded only to βNb_5_Si_3_ and only to αNb_5_Si_3_. Sn-rich Nb_3_Sn was also observed. The latter exhibited a brighter contrast under BSE imaging compared with the “normal” Nb_3_Sn. The aforementioned phases were observed in all parts of the button, the microstructure of which was coarser in the bulk and finer in the bottom. In the latter, the microstructure consisted of Nb_5_Si_3_ and Sn-rich Nb_3_Sn. In all parts of the button there was also an Nb_5_Si_3_ + Sn-rich Nb_3_Sn lamellar microstructure (indicated as eutectic in the Table S2 in the Supplemental data, see discussion) that had formed adjacent to the primary Nb_5_Si_3_ dendrites. The average composition of this lamellar microstructure was the same in the top, bulk and bottom of the button (75.5Nb-13.4Si-5.4Al-5.7Sn). No solid solution was observed by XRD and EPMA. The volume fractions of the Nb_5_Si_3_ and Nb_3_Sn were essentially the same (see Table A1 in the Appendix A).

**Heat-Treated alloy EZ7 (1500 °C/100 h):** After the heat treatment the microstructure had coarsened (Figure 3c) and consisted of αNb_5_Si_3_, βNb_5_Si_3_ and Nb_3_Sn (Table S2 in the Supplemental data and Figure 1b). There was still large scale chemical inhomogeneity of Si, the concentration of which varied between 16 and 19.3 at.% (Table S2 in the Supplemental data). There were no Sn-rich areas present in the Nb_3_Sn in which the Si concentration was reduced compared with the cast alloy. There was no change of the volume fraction of each phase compared with the cast alloy (see Table A1 in the Appendix A). Remnants of coarsened prior eutectic areas were observed.

**Figure 4:**
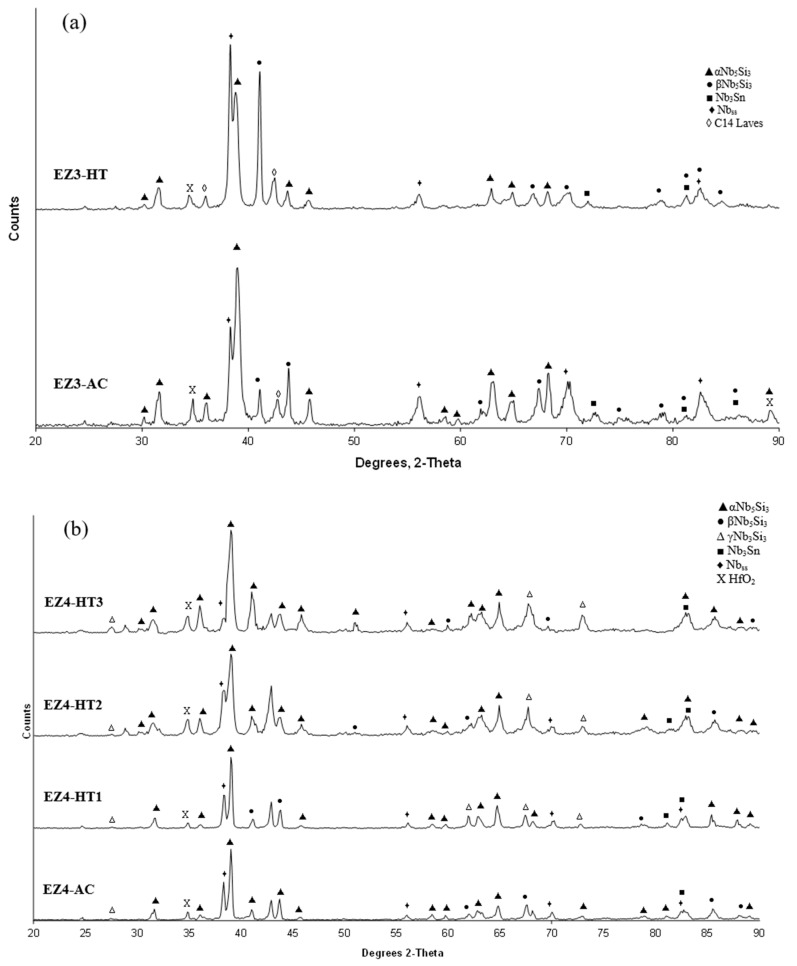
X-ray diffractograms of the as-cast and heat-treated alloys (**a**) EZ3 and (**b**) EZ4.

**Figure 5:**
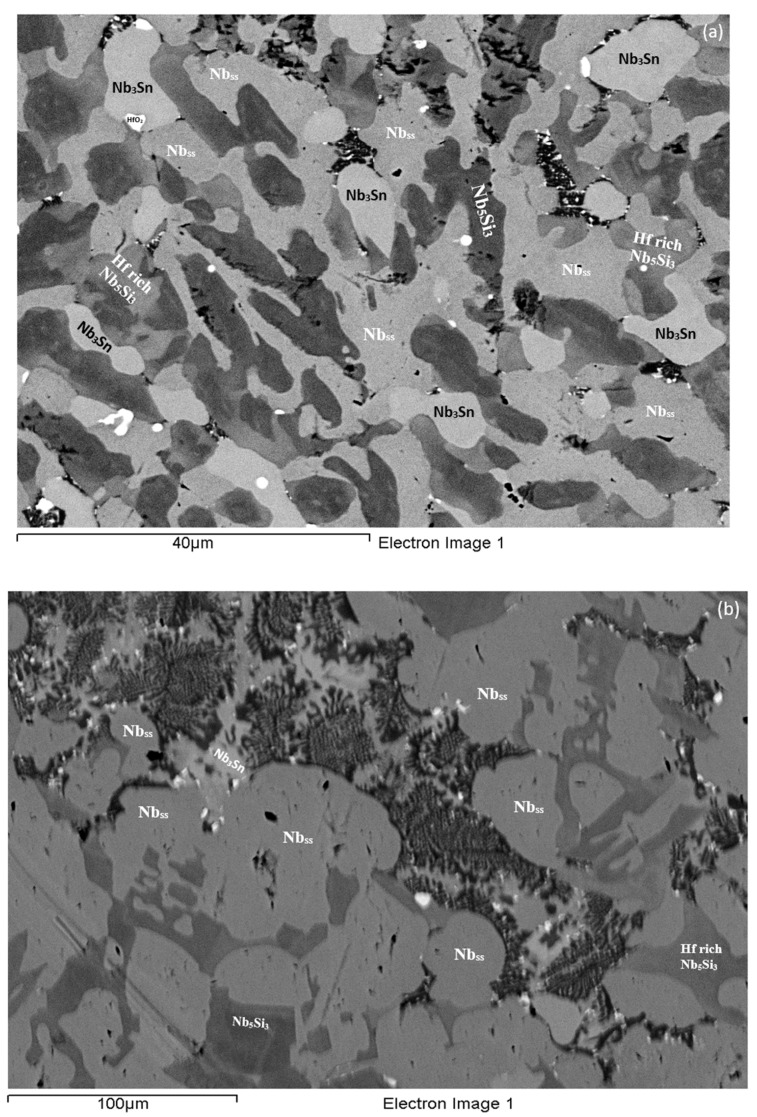

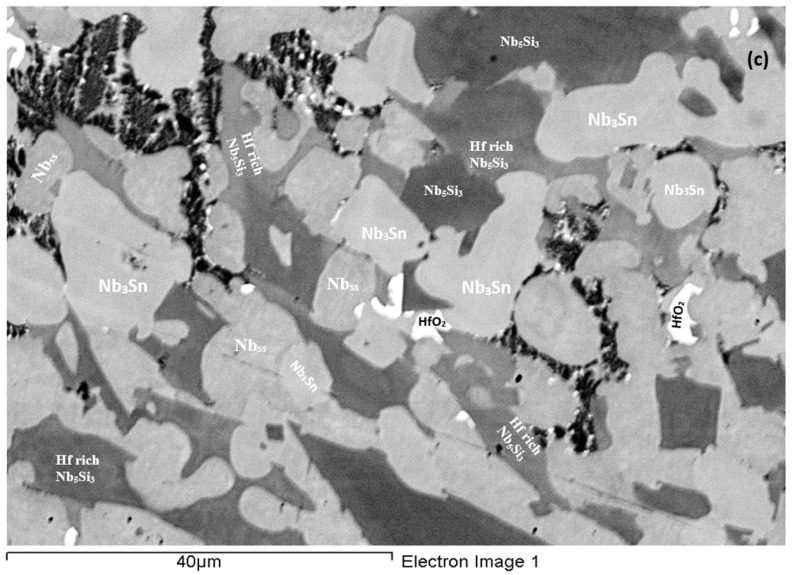
BSE images of the microstructure of the bulk (**a**) and (**c**) and bottom (**b**) of the as-cast alloy EZ3.

**As-cast alloy EZ3 (EZ3-AC):** According to the XRD data (Figure 4a) the Nb_ss_, Nb_3_Sn, αNb_5_Si_3_, βNb_5_Si_3_, HfO_2_ and C14-NbCr_2_ Laves phase were present in the microstructure. Twice as many peaks corresponded only to αNb_5_Si_3_ compared with βNb_5_Si_3_. The microstructure of the alloy is shown in Figure 5. Identification of individual phases was not always possible using BSE imaging owing to the partitioning of Hf. The parts b and c in Figure 5 are given to help the reader identify the different phases in the cast alloy. A very fine lamellar microstructure that contained the NbCr_2_ Laves phase was formed next to, or around the Nb_3_Sn, usually in the regions between the Nb_3_Sn and the Hf-rich Nb_5_Si_3_ (Figure 5b,c) but also between the Nb_ss_ and Hf rich Nb_5_Si_3_. Analysis of the composition of the Laves phase by EPMA was not possible owing to its size. The composition of the lamellar microstructure (indicated as eutectic in the Table S3 in the Supplemental data, see discussion) was the average composition of the lamellar microstructure throughout the ingot. HfO_2_ particles were observed either inside or next to the lamellar microstructure. The volume fraction of the Nb_5_Si_3_ was slightly lower than the sum of the volume fractions of the Nb_3_Sn and Nb_ss_ (see Table A1 in the Appendix A).

**Figure 6:**
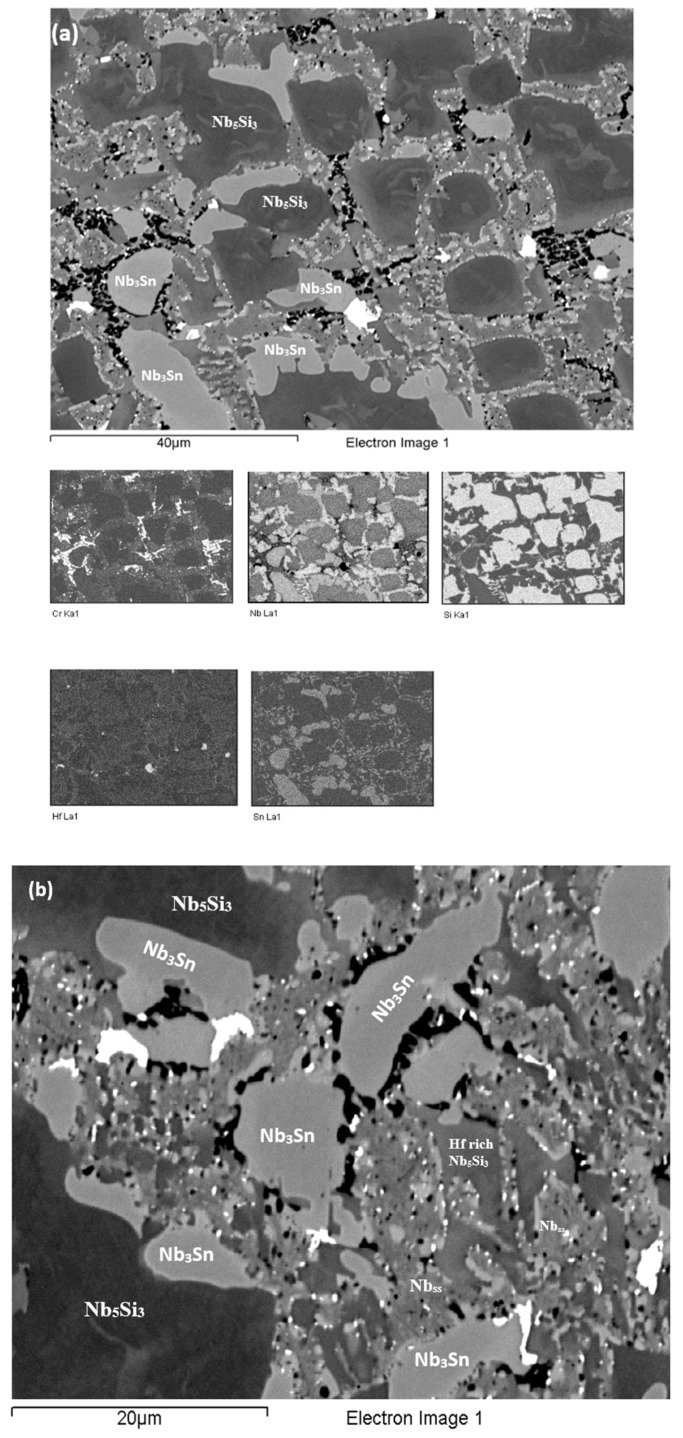
BSE images of the EZ3-HT (1200 °C/100 h) of (**a**) of the bulk with X-ray elemental maps and (**b**) details of an area with the C14-NbCr_2_ Laves phase.

**Heat-treated alloy EZ3 (1200 °C /100 h):** After this heat treatment there was still large scale chemical inhomogeneity for Si and Hf-rich areas persisted in the Nb_5_Si_3_ silicide (Table S3 in the Supplemental data). The microstructure is shown in Figure 6. Hafnia particles were mainly observed in the areas that were rich in Hf in the cast alloy. The microstructure consisted of large Nb_5_Si_3_ grains surrounded by a network of unevenly distributed Hf-rich Nb_5_Si_3_ with Nb_ss_. Submicron particles of HfO_2_ were dispersed throughout these regions (Figure 6). The Nb_3_Sn was found adjacent to these areas and the Laves phase was found next to it. The Nb_ss_ was poorer in Cr, Si and Sn compared with EZ3-AC.

The XRD data indicated the presence of Nb_ss_, Nb_3_Sn, αNb_5_Si_3_, βNb_5_Si_3,_ HfO_2_ and C14 NbCr_2_ Laves phase (Figure 4a). Approximately twice as many peaks corresponded only to αNb_5_Si_3_ compared with βNb_5_Si_3_. The solid solution was stable in the microstructure, the volume fraction of Nb_3_Sn was more than double that in the cast alloy and the volume fraction of the Nb_5_Si_3_ was reduced slightly (Table A1 in the Appendix A). The volume fraction of the NbCr_2_ Laves phase was very low. The Cr rich areas in the microstructure can be seen in the Cr map in Figure 6. The prior lamellar microstructure areas had Cr + Si + Sn = 51.0 at.%, very close to the composition of the eutectic in the binary Nb-Cr phase diagram [22]. The volume fraction of the lamellar microstructure was about 0.24. The average composition of the Laves phase gave (Cr + Si + Sn) = 59.4 at.%, but there were some analyses that gave (Cr + Si + Sn) ≈ 64 at.%.

**Figure 7:**
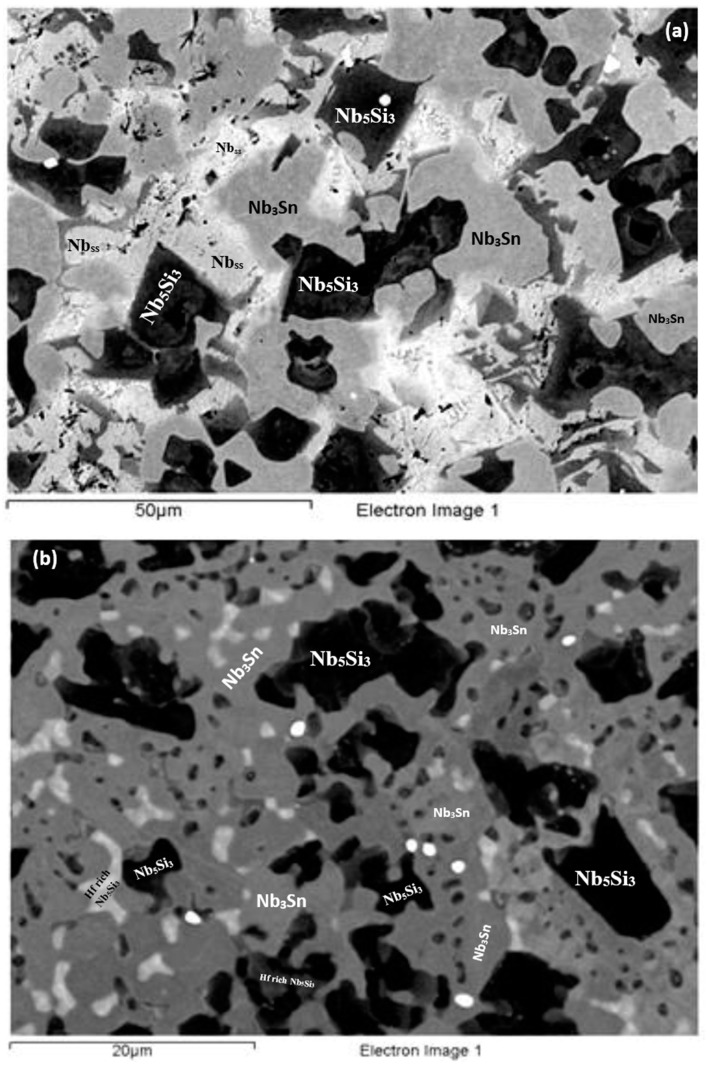

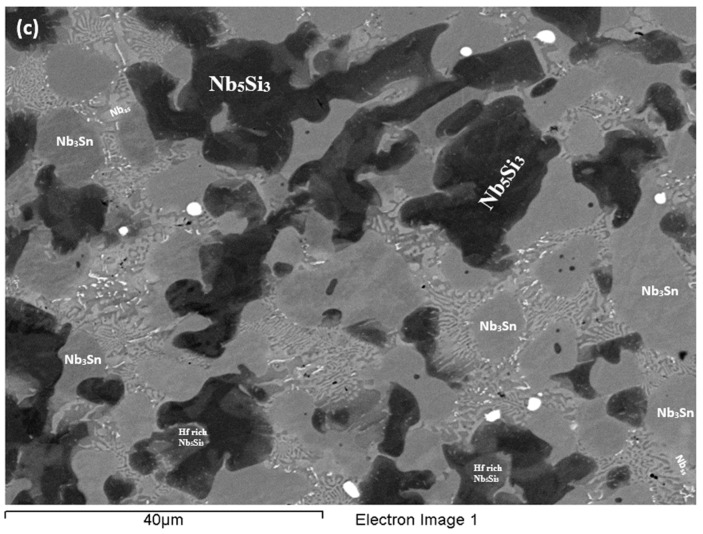
BSE images of the microstructure (**a**) in the bulk (**b**) bottom and (**c**) top of the button of the as-cast alloy EZ4.

**As-cast alloy EZ4 (EZ4-AC):** Typical images of the microstructure of the cast alloy are shown in Figure 7. The XRD data indicated the presence of Nb_ss_, αNb_5_Si_3_, βNb_5_Si_3_, γNb_5_Si_3_, HfO_2_ and Nb_3_Sn (Figure 4b). More peaks corresponded only to αNb_5_Si_3_ than only to βNb_5_Si_3_, as was the case for EZ1-AC. One very weak peak corresponded only to the γNb_5_Si_3_. The Nb_ss_ was observed only in the bulk and top of the ingot where it was formed next to the Nb_3_Sn and its volume fraction was significantly lower than those of the Nb_5_Si_3_ and Nb_3_Sn (see Table A1 in the Appendix A). Fine HfO_2_ particles were observed in all parts of the button. Hafnium rich areas were observed in the Nb_5_Si_3_ silicide and some Nb_ss_ grains (Figure 7a,c). A very fine lamellar microstructure formed adjacent to the Nb_3_Sn phase (Figure 7). It was not possible to confirm if the former was binary Nb_ss_ and Nb_5_Si_3_ or ternary Nb_ss_, Nb_5_Si_3_ and Nb_3_Sn lamellar microstructure or whether some or all of the phases in it were Hf-rich, because of the scale of the lamellae and the contrast of phases. It is for this reason that in the Table S4 in the Supplemental data the analysis data of these areas is given for eutectic with Nb_ss_ and Nb_5_Si_3_, see discussion.

**Figure 8:**
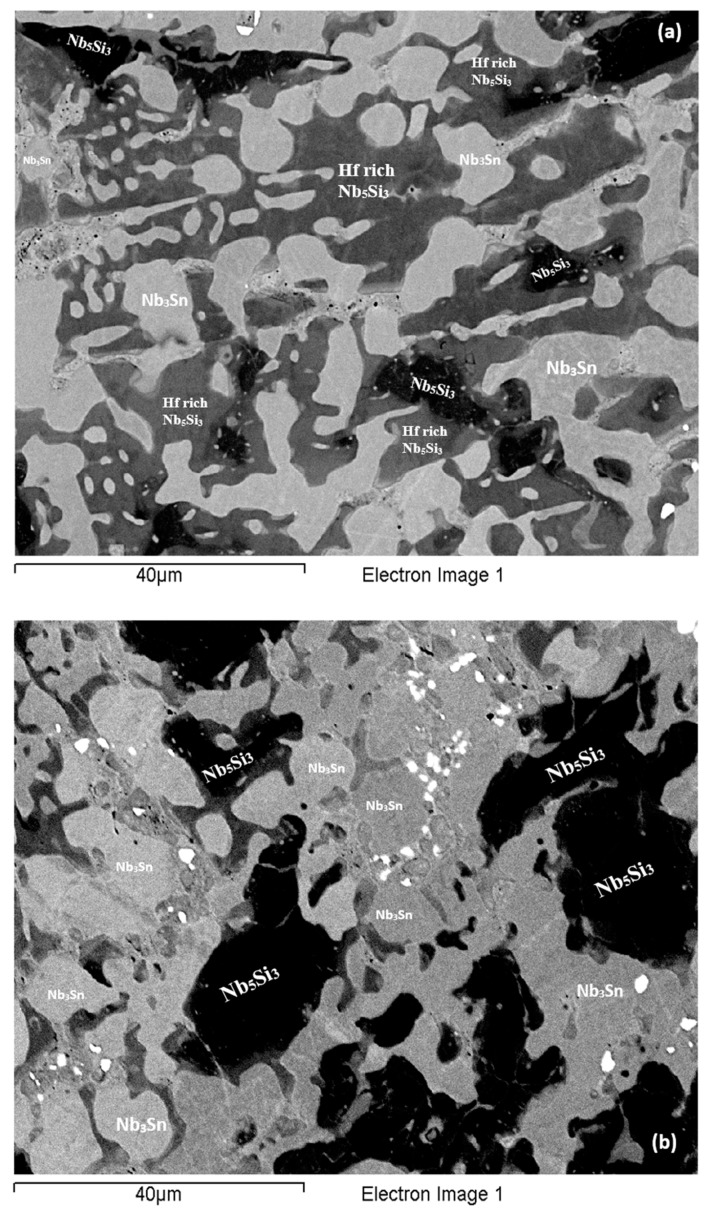

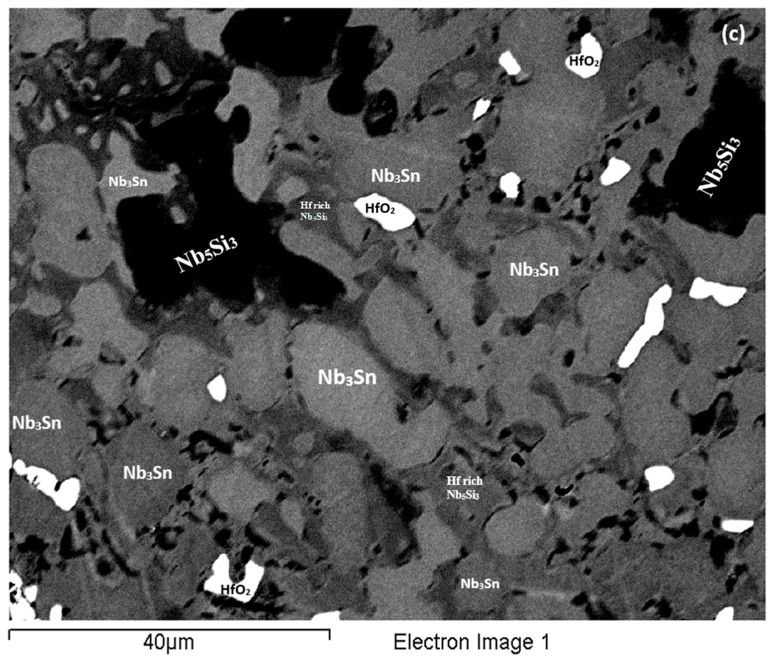
BSE images of the bulk (**a**) of the EZ4-HT (1500 °C/100 h), (**b**) of the EZ4-HT2 (1500 °C/200 h), (**c**) of the EZ4-HT3 (1500 °C/300 h).

**Heat-treated alloy EZ4-HT1 (1500 °C/100 h):** There was no large scale chemical inhomogeneity of Si after this heat treatment. The microstructure consisted of a network of intersecting Nb_5_Si_3_ and Hf-rich Nb_5_Si_3_ dendrites surrounded by Nb_3_Sn (Figure 8a). The Nb_3_Sn was poorer in Si and richer in Al compared with the cast alloy (Table S4 in the Supplemental data). Submicron Nb_3_Sn particles formed in the Nb_5_Si_3_ silicide (Figure 8a). The exact composition of these particles could not be analyzed and their identity was deduced from their imaging under BSE imaging conditions. HfO_2_ particles were observed close to the Hf-rich Nb_5_Si_3_ silicide. The XRD (Figure 4b) indicated the presence of Nb_ss_, αNb_5_Si_3_, βNb_5_Si_3_, γNb_5_Si_3_, HfO_2_ and Nb_3_Sn. The number of peaks that corresponded only to the γNb_5_Si_3_ had increased and there was no change of the number of peaks that corresponded to αNb_5_Si_3_ and βNb_5_Si_3_. Exhaustive study of the microstructure using EPMA did not confirm the existence of Nb_ss_ in EZ4-HT1. The volume fraction of the Nb_5_Si_3_ had increased compared with the cast alloy (see Table A1 in the Appendix A).

**Heat-treated alloy EZ4-HT2 (1500 °C/200 h):** The alloy EZ4 was heat treated for an additional 100 h at 1500 °C to confirm the stability or not of the Nb_ss_ and of the βNb_5_Si_3_ and γNb_5_Si_3_. The same specimen that was initially heat treated for 100 h at 1500 °C (i.e., specimen EZ4-HT1) was given another 100 h at 1500 °C (and subsequently this specimen was given another 100 h heat treatment at 1500 °C (total 300 h—EZ4-HT3, see below). The XRD (Figure 4b) indicated the presence of Nb_ss_, αNb_5_Si_3_, βNb_5_Si_3_, γNb_5_Si_3_, HfO_2_ and Nb_3_Sn. The number of peaks that corresponded only to αNb_5_Si_3_, βNb_5_Si_3_ and γNb_5_Si_3_ had not changed. Thorough study of the microstructure of EZ4-HT2 using EPMA found only two areas with composition corresponding to Nb_ss_ and confirmed that a very low volume fraction of the solid solution was present in EZ4-HT2.

The typical microstructure is shown in Figure 8b. There were Hf-rich areas in Nb_5_Si_3_, and very Hf-rich Nb_5_Si_3_ was observed for the first time. In the latter the concentration of Hf was more than double that in the Hf-rich areas in Nb_5_Si_3_ (Table S4 in the Supplemental data). In this phase the Si + Sn + Al concentration was about 39.3 at.% with the Hf and Al concentration about 19.5 at.% and 5.4 at.%, respectively. The very Hf-rich Nb_5_Si_3_ phase was surrounded by two phase regions consisting of the Nb_ss_ and Hf-rich Nb_5_Si_3_. The submicron particles that were observed in the Nb_5_Si_3_ silicide after the first heat treatment at 1500 °C (EZ4-HT1) were almost non-existent after 200 h of heat treatment. The Nb_3_Sn was poorer in Si and richer in Al compared with EZ4-AC.

Despite the fact that the compositions of Nb_3_Sn, Nb_5_Si_3_ and Hf-rich Nb_5_Si_3_ in EZ4-HT1 and EZ4-HT2 were similar (Table S4 in the Supplemental data) the microstructure of EZ4-HT2 was significantly altered. There was a change in the volume fraction of the phases; that of Nb_3_Sn had increased to about 0.605 and that of Nb_5_Si_3_ had decreased to about 0.395 (see Table A1 in the Appendix A). Remnants of the prior eutectic were not observed.

**Heat-treated alloy EZ4-HT3 (1500 °C/300 h):** The microstructure of EZ4-HT3 was essentially the same as that of EZ4-HT2. The XRD (Figure 4b) indicated the presence of Nb_ss_, αNb_5_Si_3_, βNb_5_Si_3_, γNb_5_Si_3_, HfO_2_ and Nb_3_Sn. The number of peaks that corresponded only to αNb_5_Si_3_, βNb_5_Si_3_ and γNb_5_Si_3_ had not changed compared with EZ4-HT2. Thorough examination of the microstructure using EPMA did not find any evidence for the solid solution. It was concluded that if there was solid solution present in EZ4-HT3, its volume fraction would be extremely low.

Data for the chemical composition of the phases is given in Table S4 in the Supplemental data and the typical microstructure is shown in Figure 8c. The EPMA data confirmed that the microstructure consisted of Nb_3_Sn, Nb_5_Si_3_, Hf-rich Nb_5_Si_3_, very Hf-rich Nb_5_Si_3_ and HfO_2_. As was the case in EZ4-HT2, the submicron Nb_3_Sn particles that had been observed in the Nb_5_Si_3_ silicide in EZ4-HT1 were no longer present in EZ4-HT3. The HfO_2_ particles were much more abundant and coarser especially in the regions that were closer to the edges of the specimen. The volume fraction of the phases was the same as that in EZ4-HT2 (see Table A1 in the Appendix A).

### 3.1. Density, Hardness and Lattice Parameter of Nb_ss_

Data for the density, hardness and % area of phases for the as-cast and heat treated alloys is summarized in the Table A1 in the Appendix A, and data for the lattice parameter of the Nb solid solution is given in the Table A2 in the Appendix A. The latter includes data for the alloy Nb-18Si-5Sn (alloy NV9 in [14]). The lattice parameter of the Nb_ss_ was lower than that of pure Nb (3.3007 Å) with the exception of the solid solution in the heat treated alloy EZ1, and increased after heat treatment (see Table A2 in the Appendix A).

The data in Table A1 in the Appendix A shows (a) that the density of all alloys was less than 8.4 g/cm^3^ and that the alloy EZ7 had the lowest density, (b) that the hardness of the alloys in the heat treated condition was lower than in the as-cast condition with the exception of the alloy EZ7 and (c) that the volume fraction of the Nb_3_Sn phase was high in the alloys that contained Al. The solid solution was not stable in the alloy EZ7 and (it is highly likely that it was not stable) in the alloy EZ4.

The hardness of the Nb_5_Si_3_ in the alloys was lower than that of unalloyed Nb_5_Si_3_ (1360 HV [14]) with the exception of the cast alloy EZ1 where it was essentially the same, and the hardness of the alloyed Nb_3_Sn was significantly higher than that of the unalloyed Nb_3_Sn (450 HV [14]).

### 3.2. Oxidation

All the alloys exhibited pest oxidation. The alloys EZ4 and EZ7 oxidized very rapidly and in less than 100 h had gained weights in excess of the weight measurement capability of the instrument used for the experiments. The specimen of the alloy EZ4 was converted into powders, and that of the alloy EZ7 broke into many small angular pieces. The specimen of the alloy EZ1 oxidized following linear oxidation kinetic with k_l_ = 9 × 10^−7^ g·cm^−2^·s^−1^ and after 100 h was converted into powders. The specimen of the alloy EZ3 also followed linear kinetics with k_l_ = 8.5 × 10^−7^ g·cm^−2^·s^−1^ and after 100 h formed powder and a smaller cubic solid core.

## 4. Discussion

### 4.1. Macrosegregation of Si

**Figure 9:**
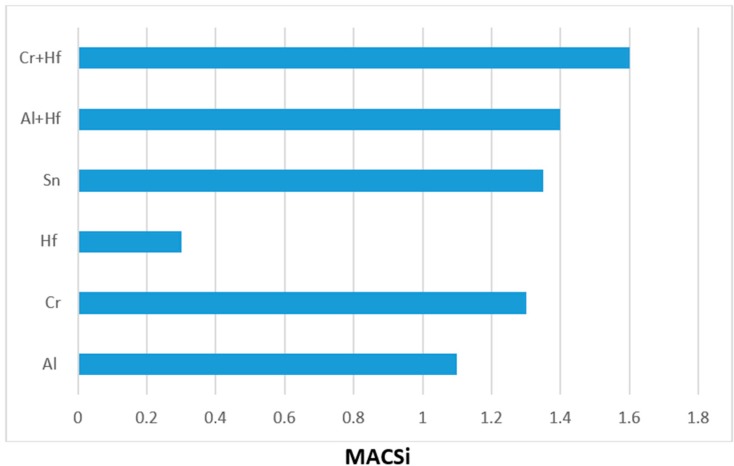
Effect of alloying additions on the macrosegregation of Si (MACSi).

Macrosegregation is common in alloys that are prepared using arc melting with water cooled crucibles and has been reported in many Nb-silicide based alloys [21]. There was macrosegregation of Si (MACSi) in all the alloys and the chemical inhomogeneity of Si persisted in the heat treated microstructures of the alloys with the exception of the alloy EZ4. The effects of specific element additions individually and simultaneously on the macrosegregation of Si after casting were separated by comparing the data for different alloys. Figure 9 summarizes the effects of Al, Cr, Hf and Sn individually and Al + Hf and Cr + Hf simultaneously on the macrosegregation of Si after casting. Figure 9 shows that among the single element additions Hf had the weakest and Sn the strongest effect on MACSi, and that the synergy of Cr and Hf had the strongest effect on MACSi.

The effect of the alloying additions of Al, Cr, Hf and Sn on the macrosegregation of Si in the alloys EZ1, EZ7, EZ3 and EZ4 also was studied using the parameters discussed in [21]. Table 2 shows that different parameters controlled MACSi when Hf and Sn were in synergy with Al or Cr. In the former case the increase of MACSi was associated with the increase of the parameters T_m_^sp^, ΔH_m_^sp^, ΔH_m_^alloy^/T_m_^alloy^ and in the latter with a decrease of the parameters T_m_^alloy^, ΔH_m_^alloy^, ΔH_m_^sd^ and T_m_^sd^, in agreement with [21].

### 4.2. Microstructures

#### 4.2.1. Primary Phase

In all the as-cast buttons of the alloys of this study the Nb_5_Si_3_ was the primary phase. The XRD data (Figure 1 and Figure 4) indicated that both αNb_5_Si_3_ and βNb_5_Si_3_ were present in the as-cast microstructures and after the heat treatment(s). This was also the case in the previously studied alloys without Hf (Nb-18Si-5Sn, alloy NV9 in [14]) and Sn (Nb-18Si-5Cr-5Hf and Nb-18Si-5Al-5Hf, respectively alloys YG1 and YG2 in [17]).

Which type of silicide (meaning βNb_5_Si_3_ or αNb_5_Si_3_) was the primary silicide in the as-cast buttons of the alloys EZ1, EZ7, EZ3 and EZ4? To answer this question, we need to consider the data in Table A3 in the Appendix A, which summarizes data about the type of Nb_5_Si_3_ in as-cast and heat treated Nb-silicide based alloys of different size (weight) buttons, suction cast bars and directionally solidified (DS) alloys prepared using optical float zone melting (OFZ) or liquid-metal cooled directional solidification. Large button means weight of about 0.6 kg or higher, small button means weight of about 0.03 kg or lower, suction cast means bars cast in water cooled copper crucibles with diameter of 8 mm or lower and OFZ means bars grown using optical floating zone melting with diameter about 10 mm or lower.

The Table A3 in the Appendix A shows (i) that there is only one systematic study where an alloy of a specific composition, namely the alloy CM1, was studied using the full range of experimental techniques, from 0.01 g small buttons to 6 mm and 8 mm diameter suction cast bars, to 0.6 kg large buttons to 10 mm diameter OFZ bars, (ii) that whether the βNb_5_Si_3_ does not transform to αNb_5_Si_3_ or whether the βNb_5_Si_3_ transforms to αNb_5_Si_3_ completely or partially in an as-cast Nb-silicide based alloy depends (a) on alloy composition and (b) on solidification conditions, and that the latter depend on the size of the button, as confirmed by the systematic study of the alloy CM1 [19] and (iii) that whether after the heat treatment of a given alloy the βNb_5_Si_3_ transforms to αNb_5_Si_3_ completely or partially depends on the alloy composition and the heat treatment conditions (temperature and duration of heat treatment). Furthermore, the results for the alloy CM1 that are summarized in Table A3 in the Appendix A show that if only large buttons of this alloy had been studied, the conclusion that the αNb_5_Si_3_ was the primary phase in CM1 would have been erroneous and misleading.

Areas that correspond to the βNb_5_Si_3_ and αNb_5_Si_3_ silicides appear in some of the liquidus projections that have been proposed for the Nb-Ti-Si system. They also appear in a liquidus projection for the Nb-Si-Sn system [23] (see Supplemental data). There are 7 different versions of the projection for the former system [24,25,26,27,28,29,30] and one for the latter. In the case of the Nb-Ti-Si system some projections are experimental and some are calculated. In some the type of Nb_5_Si_3_ is not specified, others indicate that only the β(Nb,Ti)_5_Si_3_ could form from the melt and others that the β(Nb,Ti)_5_Si_3_ or the α(Nb,Ti)_5_Si_3_ could form from the melt depending on alloy composition. In the latter case, in some liquidus projections the area of the αNb_5_Si_3_ is very large and in others is very small.

Considering the data in Table A3 in the Appendix A for the type of Nb_5_Si_3_ formed in as-cast Nb-silicide based alloys, the results of the systematic study of the alloy CM1 [19], the liquidus projection by Sun et al. [23] and Figure S1 in the Supplemental data, it is concluded (i) that it is highly unlikely that the αNb_5_Si_3_ was the primary phase in the alloy Nb-18Si-5Sn (alloy NV9 in [14]), and (ii) that the experimental data for the as-cast and heat treated alloy NV9 in [14] does not support the proposal by Sun et al. [23] for the invariant reaction L → (Nb) + A15 + αNb_5_Si_3_. Instead, the experimental data points to the eutectic reaction L → (Nb) + βNb_5_Si_3_.

The above discussion, the discussion of the Nb-Si-Sn liquidus projection in the Supplemental data and the microstructural data for the alloys EZ1, EZ7, EZ3 and EZ4 would suggest that the primary phase in the large buttons of all these alloys was the βNb_5_Si_3_, which then partially transformed to αNb_5_Si_3_ during solidification. The experimental data also would suggest that the βNb_5_Si_3_ to αNb_5_Si_3_ transformation was not completed, even after the longest heat treatment. The co-existence of both αNb_5_Si_3_ and βNb_5_Si_3_ in the as-cast and heat treated microstructures of the alloys EZ1, EZ3 and EZ4 also could be attributed to Hf (a group IV element) and Sn having the same effect as Ti (a group IV element) did with Sn in the alloy Nb-24Ti-18Si-5Sn (NV6 in [14]), i.e., they enhanced the aforementioned transformation. The microstructure of EZ1-AC was finer compared with that of the as-cast alloy Nb-18Si-5Sn (NV9). This effect has been attributed to the addition of Hf, and is in agreement with [16].

In all the alloys of this study the Nb_3_Si was not observed. This is consistent with Sn suppressing this silicide [14] and would suggest that this effect of Sn is so strong that eliminates the effect of Hf, which stabilized the Nb_3_Si in ternary alloys without Sn [15,16]. The Nb_3_Sn was stable in all the alloys of this study. The latter effect may be attributed to the concentration of Sn being higher than 2 at.% in these cast and heat treated alloys (see Tables S1–S4 in the Supplemental data). In the Nb_3_Sn, the Si + Sn or Si + Sn + Al sums did not vary significantly between the alloys (Table 3).

#### 4.2.2. Eutectics

Suppression of Nb_3_Si in Nb-silicide based alloys is accompanied by the suppression of the L → Nb_ss_ + Nb_3_Si eutectic. The latter can be replaced by the L → Nb_ss_ + βNb_5_Si_3_, depending on alloy composition. For example, the addition of Al in the alloy Nb-24Ti-18Si-5Al (alloy KZ7 in [31]) suppressed the Nb_3_Si and stabilized the Nb_ss_ + βNb_5_Si_3_ eutectic. Lamellar microstructures reminiscent those of eutectics were observed in the alloys EZ1, EZ7, EZ3 and EZ4. In the alloys EZ1, EZ4, these lamellar microstructures had Si + Sn and Si + Sn + Al concentrations, essentially the same with that in the alloy Nb-18Si-5Sn (NV9) (Table 3).

In the as-cast alloy EZ1 the lamellar microstructure was observed in all parts of the button but the A15-Nb_3_Sn was observed only in the bulk of the button. Furthermore, the average composition of this microstructure did not vary along the button and was essentially the same as that of the Nb_ss_ + βNb_5_Si_3_ eutectic in the as-cast button of the alloy Nb-18Si-5Sn (NV9) (see Figure S1b in the Supplemental data). The microstructures in Figure 2 would suggest that the lamellar microstructure observed in all parts of the as-cast button of the alloy EZ1 was the binary Nb_ss_ + βNb_5_Si_3_ eutectic and not the ternary Nb_ss_ + βNb_5_Si_3_ + A15-Nb_3_Sn eutectic.

The Nb_ss_ was suppressed when Al was added in the alloy EZ7 and the binary βNb_5_Si_3_ + Sn rich A15-Nb_3_Sn eutectic was formed in all parts of the large button. In the alloy EZ3 the addition of Cr promoted the formation of C14-NbCr_2_ Laves phase and did not suppress the Nb_ss_. The latter two phases participated in a lamellar microstructure with Hf-rich Nb_5_Si_3_ that was observed in all parts of the large button. The composition of this microstructure after the heat treatment moved very close to that of the Nb + NbCr_2_ eutectic in the Nb-Cr binary. The composition of the Laves phase in this alloy was in agreement with the literature about Laves phases in Nb-silicide based alloys [5]. It is suggested that the lamellar microstructure in the as-cast large button of the alloy EZ3 was ternary Nb_ss_ + NbCr_2_ + Hf-rich Nb_5_Si_3_ eutectic.

In the as-cast large button of the alloy EZ4 the Nb_ss_ was suppressed in the bottom but not in the bulk and top of the button where a very fine lamellar microstructure was observed. The partitioning of Hf in the microstructure did not allow us to determine whether this microstructure consisted of two or three phases. Given that binary Nb_ss_ + Nb_5_Si_3_ and Nb_5_Si_3_ + Nb_3_Sn eutectics were formed respectively in the alloys EZ1 and EZ7, it is suggested that when Al and Hf were simultaneously present in the alloy EZ4 a ternary Nb_ss_ + Nb_5_Si_3_ + Nb_3_Sn eutectic formed only in the parts of the button where the solid solution was not suppressed, and that the synergy of Al and Hf suppressed the binary Nb_5_Si_3_ + Nb_3_Sn eutectic (no lamellar microstructure was observed in the bottom of the large button of EZ4 where only Nb_5_Si_3_ and Nb_3_Sn were formed).

#### 4.2.3. Solidification

The formation of the Nb_3_Sn and the eutectic, respectively in the alloys EZ1 and EZ4 was sensitive to cooling rate. The Nb_3_Sn formed only in the bulk of EZ1-AC. In the latter, as the βNb_5_Si_3_ formed, the melt became leaner in Si and Hf, and richer in Sn. Thus, close to βNb_5_Si_3_ the melt reached a composition with Si + Sn ≈ 17 at.% (Table 3) and the Nb_3_Sn formed; the melt continued to become leaner in Hf, and leaner in both Si and Sn and eventually reached the eutectic composition, leading to the eutectic reaction L → Nb_ss_ + βNb_5_Si_3_. In the top and bottom of EZ1-AC no Nb_3_Sn was formed. The scale of the microstructure was finer in the bottom of EZ1-AC. Compared with the alloy Nb-18Si-5Sn (NV9) [14], where the Nb_3_Sn was present everywhere in the cast microstructure, the formation of Nb_3_Sn only in the bulk of EZ1-AC would suggest that in the presence of Hf the formation of Nb_3_Sn was strongly affected by (became sensitive to) cooling rate. It is suggested that the solidification path was L → L + βNb_5_Si_3_ → L + βNb_5_Si_3_ + Nb_3_Sn → βNb_5_Si_3_ + Nb_3_Sn + (Nb_ss_ + βNb_5_Si_3_) eutectic and L → L + βNb_5_Si_3_ → βNb_5_Si_3_ + (Nb_ss_ + βNb_5_Si_3_) eutectic respectively in the bulk, and top and bottom parts of EZ1-AC, with βNb_5_Si_3_ transforming to αNb_5_Si_3_ during solid state cooling.

In the top and bulk of the large button of the alloy EZ4, as the primary βNb_5_Si_3_ formed the melt became leaner in Si and richer in Al and Sn. When the melt concentration reached Si + Sn + Al ≈ 19.0 at.% (Table 3) the Nb_3_Sn formed. As the melt became leaner in Al and Sn and richer in Hf the Nb_ss_ + Hf-rich Nb_5_Si_3_ eutectic formed. The βNb_5_Si_3_ transformed to αNb_5_Si_3_ during solid state cooling of the large button. In the bottom of the button the Nb_ss_ + Hf-rich Nb_5_Si_3_ eutectic was suppressed as the partitioning of solutes was affected by the high cooling rate(s) there. It is suggested that the solidification path was L → L + βNb_5_Si_3_ → L + βNb_5_Si_3_ + Nb_3_Sn → βNb_5_Si_3_ + Nb_3_Sn + eutectic + αNb_5_Si_3_ and L → L + βNb_5_Si_3_ → L + βNb_5_Si_3_ + Nb_3_Sn → βNb_5_Si_3_ + Nb_3_Sn + αNb_5_Si_3_, respectively in the top and bulk, and the bottom of EZ4-AC (see earlier discussion about the eutectic in EZ4-AC).

When the Nb_ss_ was suppressed in the alloy EZ7 by the synergy of Al, Si and Sn and the Nb_3_Sn was formed, the latter replaced the solid solution in the eutectic with Nb_5_Si_3_. The addition of Al affected the partitioning of Sn between βNb_5_Si_3_ and Nb_3_Sn during solidification. As the primary βNb_5_Si_3_ formed, the melt became leaner in Si and richer in Al and Sn, and when it reached a composition with Si+Sn+Al ≈ 20 at.% (Table 3) the Sn-rich Nb_3_Sn formed around the silicide. As the temperature decreased further the melt finally reached the Nb_5_Si_3_-Nb_3_Sn eutectic composition and the eutectic formed. It is suggested that the solidification path of EZ7-AC was L → L + βNb_5_Si_3_ → L + βNb_5_Si_3_ + Nb_3_Sn → βNb_5_Si_3_ + Nb_3_Sn + (Nb_5_Si_3_ + Nb_3_Sn) eutectic with some βNb_5_Si_3_ transforming to αNb_5_Si_3_ during solid state cooling.

In the alloy EZ3 the solubility of Cr in the primary Nb_5_Si_3_ was negligible (see Table S3 in the Supplemental data), thus as the βNb_5_Si_3_ formed the melt became richer in Cr and Sn and leaner in Si and Hf. When the melt reached a composition of Si + Sn ≈ 18 at.% (Table 3) the Nb_3_Sn phase formed. The Cr, Hf and Si were rejected into the melt, which became rich in these elements. When the Si/Sn ratio in the melt reached ≈ 0.3 (Table 3) the Nb_ss_ formed. Then the melt became richer in Si and when its composition approached that of the eutectic in the Nb-Cr binary, a eutectic that contained the C14-NbCr_2_ Laves phase and the Nb_ss_ grew. As the partitioning of solutes occurred between the solidifying intermetallics and the solid solution, the aforementioned eutectic formed in between these phases. It is suggested that the solidification path of the alloy EZ3 was L → L + βNb_5_Si_3_ → L + βNb_5_Si_3_ + Nb_3_Sn → L + βNb_5_Si_3_ + Nb_3_Sn + Nb_ss_ → L + βNb_5_Si_3_ + Nb_3_Sn + eutectic → βNb_5_Si_3_ + Nb_3_Sn + eutectic + αNb_5_Si_3_ (for the eutectic see earlier discussion in this section).

#### 4.2.4. Composition of Phases and Heat Treated Microstructures

**Figure 10:**
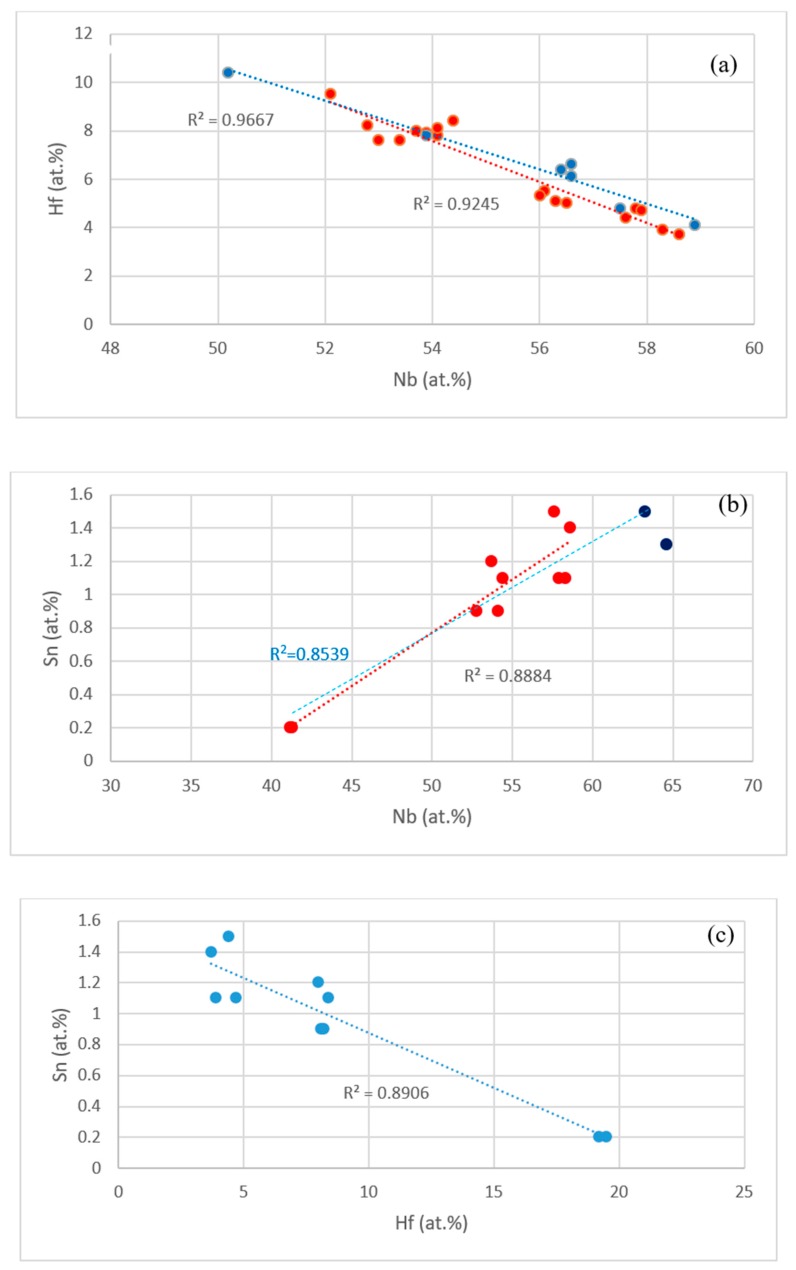
(**a**) Hf (ordinate) versus Nb (abscissa) in Nb_5_Si_3_ and Hf rich Nb_5_Si_3_ for the alloys EZ1, EZ3, EZ4 (red data points) and Nb-18Si-5Cr-5Hf (YG1 [17]) and Nb-18Si-5Al-5Hf (YG2 [17]) (blue data points), (**b**) Sn (ordinate) versus Nb (abscissa) in Nb_5_Si_3_ in the Al containing alloys EZ4 and EZ7. Data for alloy EZ4 is shown in red. All data R^2^ = 0.8539, data for the alloy EZ4 R^2^ = 0.8884, (**c**) Sn (ordinate) versus Hf (abscissa) in Nb_5_Si_3_ in the Al containing alloy EZ4.

**Figure 11:**
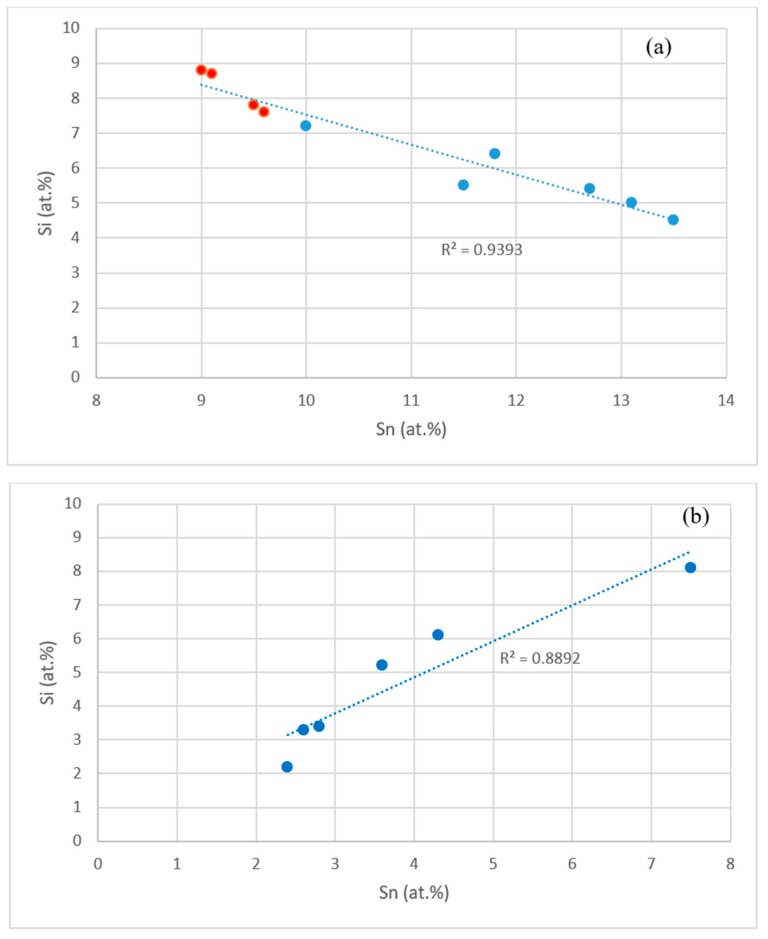
(**a**) Si (ordinate) versus Sn (abscissa) in Nb_3_Sn in alloys without Al, red data points for the alloy NV9 [14], (**b**) Sn (ordinate) versus Hf (abscissa) in Nb_ss_ in alloys without Al.

The data in the Tables S1–S4 in the Supplemental data and in [14,17] shows that the concentrations of specific elements in Nb_5_Si_3_, Nb_3_Sn and Nb_ss_ were related, as shown in the Figure 10 and Figure 11. For example, the concentrations of Hf and Sn in Nb_5_Si_3_, respectively decreased and increased with the concentration of Nb, see Figure 10a,b, and these trends are in agreement with the relationship between the Sn and Hf concentrations in Nb_5_Si_3_ shown in Figure 10c. The Si concentration in Nb_3_Sn decreased with increasing Sn in the alloys without Al, see Figure 11a. Similar relationships (not shown) exist between Al and Si, and Al and Sn in Nb_3_Sn, meaning the Si or Sn concentration decreases with increasing Al concentration in the Nb_3_Sn. In the Nb_ss_, the Sn concentration increased with increasing Hf in the alloys without Al, see Figure 11b.

Comparison of the data for EZ1-AC with that for the as-cast alloy Nb-18Si-5Sn (NV9) [14] shows (i) that in the Nb_ss_ the Si + Sn content was higher (8.2 to 10 at.% in EZ1-AC vs. 5.9 at.% in NV9-AC) and (ii) that the Si/Sn ratio was the same (≈0.3) (Table 3). This has been attributed to the higher concentration of both Si and Sn in Nb_ss_ in the presence of Hf, and the trend for the Si concentration to decrease and that of Sn to increase with increasing Hf concentration in the solid solution (see Table S1 in the Supplemental data and Figure 11b). Regarding the Nb_3_Sn, the Si + Sn content was similar to that in the cast Nb-18Si-5Sn (NV9-AC, see Table 3) but the Si/Sn ratio was lower (0.43 to 0.72 vs. 0.98 in NV9-AC). This has been attributed to the Si and Sn concentrations in Nb_3_Sn, respectively decreasing and increasing in the presence of Hf. Finally, the Si + Sn content in Nb_5_Si_3_ was higher in EZ1-AC compared with NV9-AC (Table 3). This has been attributed to the higher concentrations of both Si and Sn in Nb_5_Si_3_ in the presence of Hf. The data for EZ1-AC would thus suggest (i) that the concentrations of Si and Sn in Nb_ss_, Nb_3_Sn and Nb_5_Si_3_ respectively decrease and increase with increasing Hf concentration in these phases, and (ii) that when the Sn is in synergy with Hf the formation of Nb_ss_ and Nb_3_Sn during solidification is controlled respectively by the Si/Sn ratio and the Si + Sn sum. In the Nb_5_Si_3_ in the alloy EZ1 the Si + Sn concentration was ≈38.4 at.% and did not change after the heat treatments, indicating that this was the equilibrium Si + Sn concentration in Nb_5_Si_3_ in this alloy. The ratio (Nb + Hf)/(Si + Sn) = 1.6 was very close to the stoichiometric composition of the Nb_5_Si_3_ phase (Nb/Si = 1.67) in the binary Nb-Si [18,22]. Table 3 shows that the Nb_ss_, Nb_5_Si_3_ and Nb_3_Sn phases in the alloys EZ1, EZ7, EZ3 and EZ4 formed with specific Si/Sn or Si/(Sn + Al) ratios and Si + Sn and Si + Sn + Al sums.

If we were to consider (i) the available phase equilibria data for the Nb-Si-Sn and Nb-Si-Al ternary systems, (ii) that both Nb_3_Sn and Nb_3_Al are A15 compounds [22] and (iii) the Si + Sn and Si/Sn, and Si + Sn + Al and Si/(Sn+Al) values in Nb_3_Sn, respectively in the alloys Nb-18Si-5Sn (NV9) [14] and EZ7 (Table 3) and treat Sn and Al as equivalent, then an alloy with the actual composition of EZ7-AC, namely Nb-19Si-9(Sn + Al), would be in two phase equilibrium between the Nb_5_Si_3_ and Nb_3_Sn phases (a) in the 1600 °C isothermal section of the Nb-Si-Sn system [32], (b) in the 1400 °C isothermal sections of the Nb-Si-Al system in [33,34] and (c) in the 1000 °C isothermal section of the Nb-Si-Al system proposed by Zhao et al. [35]. If we were to treat Sn and Al, and Nb and Hf as equivalent in the alloy EZ4, then the phases present in the heat treated alloy EZ4 can be explained by considering available phase equilibrium data. An alloy with composition 72.6(Nb + Hf)-19.8Si-7.6(Sn + Al) (=EZ4-HT1) (a) would be in two phase equilibrium (Nb_5_Si_3_ and Nb_3_Sn or Nb_5_Si_3_ + Nb_3_Al) respectively (i) in the 1600 °C isothermal section of the Nb-Si-Sn system [32] and (ii) in the 1000 °C isothermal section of Nb-Si-Al [36] or (b) just at the border between the two phase Nb_5_Si_3_ + Nb_3_Al and three phase Nb_ss_ + Nb_5_Si_3_ + Nb_3_Al areas (iii) in the 1400 °C isothermal sections of Nb-Si-Al by Brukl et al. [33], Pan et al. [34] and Shao [36] and (iv) in the 1000 °C isothermal sections of Nb-Si-Al by Zhao et al. [35] and Shao [36]. An alloy with composition 73.6(Nb + Hf)-18.9Si-7.5(Sn + Al) (=EZ4-HT2) or 73.4(Nb + Hf)-18.6Si-8(Sn + Al) (=EZ4-HT3) (c) would be just at the border between the two phase Nb_5_Si_3_ + Nb_3_Sn and three phase Nb_ss_ + Nb_5_Si_3_ + Nb_3_Sn areas in the 1600 °C isothermal section of the Nb-Si-Sn system [32] and (d) just at the border between the two phase Nb_5_Si_3_ + Nb_3_Al and three phase Nb_ss_ + Nb_5_Si_3_ + Nb_3_Al areas (v) in the 1000 °C isothermal sections of Nb-Si-Al by Zhao et al. [35] and Shao [36] and (vi) in the 1400 °C isothermal sections of Nb-Si-Al by Brukl et al. [33], Pan et al. [34] and Shao [36].

Aluminium and Sn atoms substitute for Si atoms in the Nb_5_Si_3_ silicide. Brukl et al. [33] and Murakami et al. [37] reported that the Al solubility is almost zero in Nb_5_Si_3_, while Pan et al. [34] gave a solubility of ≈10 at.%. Zhao et al. [35] reported the solubility of Al in αNb_5_Si_3_ to be ≈8 to 12 at.% depending on temperature and Zelenitsas and Tsakiropoulos [38,31] reported that the concentrations of Al in αNb_5_Si_3_ was in the range 2 to 3.8 at.%, and was lower than that in the βNb_5_Si_3_ which was in the range 3.3 to 3.8 at.% in the alloy Nb-24Ti-18Si-5Al. In the alloy EZ7 the concentration of Al in Nb_5_Si_3_ was similar to that reported in [31]. The Sn concentration in the Nb_5_Si_3_ was ≈1.5 at.% in EZ7-AC and educed after the heat-treatment to ≈1.4 at.%. These values are very close to the Sn concentration in the Nb_5_Si_3_ in the alloys EZ1 and Nb-18Si-5Sn (NV9), indicating that the addition of Al did not change the solubility of Sn in the Nb_5_Si_3_ silicide.

The increase in the Hf concentration in Hf-rich areas of Nb_5_Si_3_ in the alloy EZ4 during prolonged heat treatment at 1500 °C was attributed to the strong partitioning of Hf to the latter silicide. Indeed, the 1500 °C isothermal section proposed by Bewlay et al. [39] for the Nb-Hf-Si system shows that the measured solubility of Hf in Nb_5_Si_3_ was ≈16 at.%. Yang et al. [15] calculated this solubility to be about 17.6 at.%. The higher concentration (≈19.5 at.% Hf) measured in this work in the very Hf-rich Nb_5_Si_3_ (Table S4 in the Supplemental data) would suggest that the synergy of Hf with Sn and Al increased the Hf solubility in the latter silicide. The formation of the very Hf-rich Nb_5_Si_3_ phase in the alloy EZ4 was accompanied by an increase of the Al concentration and a decrease of the Sn concentration in Nb_5_Si_3_, compared with the Hf-rich Nb_5_Si_3_. This would suggest that the concentrations of Al and Sn in Nb_5_Si_3_, respectively increase and decease with increasing Hf concentration. The average Si + Sn + Al concentration in Nb_5_Si_3_ remained essentially the same (≈37.8 at.%) after the heat treatment, which would suggest that this was the equilibrium concentration in the EZ4. This value was higher by ≈2.4 at.% compared with EZ7-HT.

The addition of Sn in the alloy Nb-18Si-5Sn (NV9 in [14]), of Hf in the alloy EZ1 and of Al in the alloy Nb-18Si-5Hf-5Al (alloy YG2 in [17]) led to the formation of a high volume fraction of eutectic with average composition 79.3Nb-20.7(Si + Sn) in NV9-AC, 78.7Me-21.3(Si + Sn) in EZ1-AC and 79Me-21(Si + Al) in YG2-AC, where Me represents transition metals in the alloy. In the alloys EZ1 and YG2 (Hf containing alloys) the eutectic was formed in all parts of the large buttons between the Nb_ss_ and Hf-rich Nb_5_Si_3_. The data would thus suggest that Si in synergy with Sn (NV9) or Sn + Hf (EZ1) or Hf + Al (YG2) stabilizes a eutectic between the solid solution and the Nb_5_Si_3_ and this eutectic, which does not exist in the equilibrium Nb-Si system, occurs at about 79 at.% sd element and 21 at.% sp element additions. The latter concentration is in agreement with the data in [7]. In the alloy EZ7 (no Hf present) where Si was in synergy with Sn and Al, the eutectic was between Nb_5_Si_3_ and Nb_3_Sn with average composition 76Me-24(Si + Sn + Al), but in the alloy EZ4, where Si was also in synergy with Sn, Al and Hf, the eutectic was again stabilized with 21.7 at.% sp element addition, in agreement with [7]. Thus, the data would suggest that Hf has a strong stabilizing effect on eutectics with Nb_ss_ and βNb_5_Si_3_, which, however, can be destabilized at high cooling rates (the eutectic was not observed in the bottom of EZ4-AC) when Hf is in synergy with Sn and Al.

#### 4.2.5. Lattice Parameter of Nb_ss_

**Figure 12:**
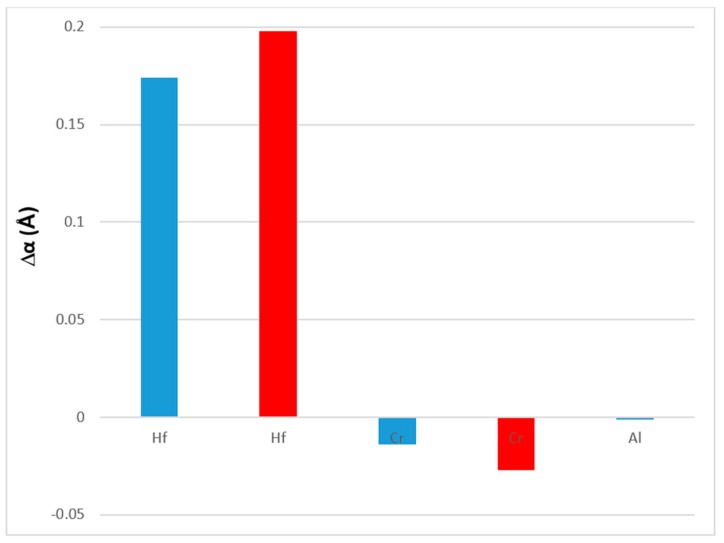
Effect of alloying addition on the change Δα (Å) of the lattice parameter of Nb_ss_. Data for the heat treated condition is shown in red.

The effect of the alloying additions of Al, Cr and Hf on the lattice parameter of the Nb_ss_ is shown in Figure 12. Comparison of the data for the alloys Nb-18Si-5Sn (NV9) and EZ1 in Table A2 in the Appendix A shows that the addition of Hf increased the lattice parameter by 0.174 and 0.198 Å in the as-cast (AC) and heat treated (HT) conditions, respectively, while comparison of the alloys EZ3 and EZ4 with EZ1 shows that the addition of Cr or Al decreased the lattice parameter, with Cr having a stronger effect than Al.

**Figure 13:**
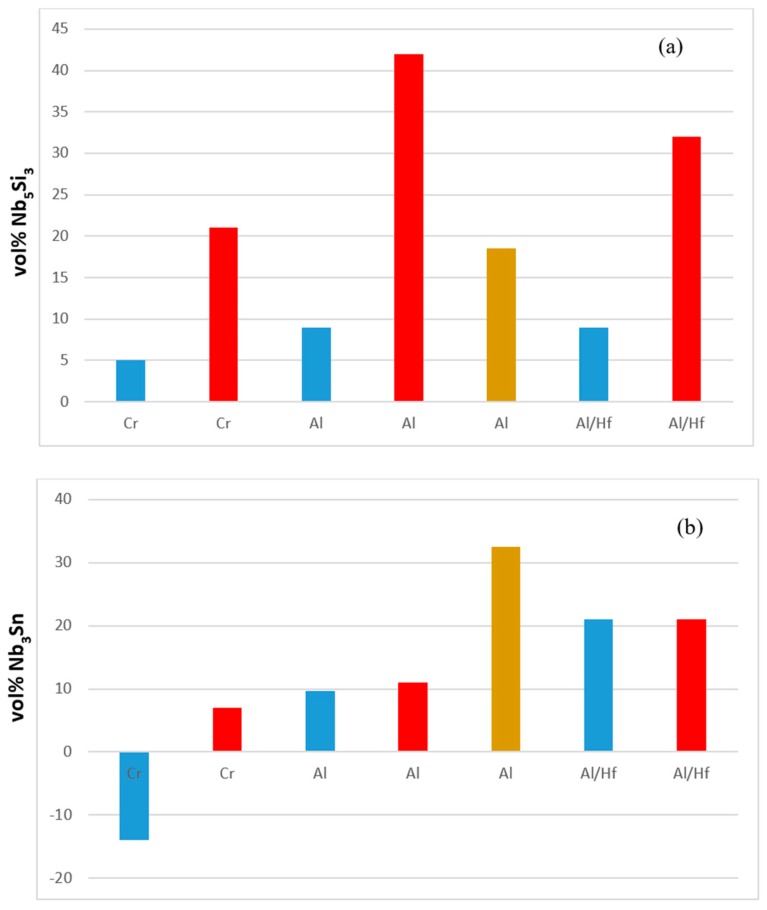
Effect of alloying addition on vol% of Nb_5_Si_3_ (**a**) and Nb_3_Sn (**b**) in the alloys, with reference the alloy EZ1. Al/Hf means Al substitutes Hf (i.e., alloy EZ7 compared with the alloy EZ1). Blue and red colour respectively for the AC and HT condition. Brown colour for longer (200 h) heat treatment.

The Nb_5_Si_3_ and Nb_3_Sn were stable in all alloys (Table 1 and Tables S1–S4 in the Supplemental data and Table A1 in the Appendix A). Figure 13 shows the effect of Al, Cr and Hf on the volume fractions of the two intermetallic phases. Aluminium had a strong effect on their volume fractions particularly after the heat treatment.

### 4.3. Hardness

**Figure 14:**
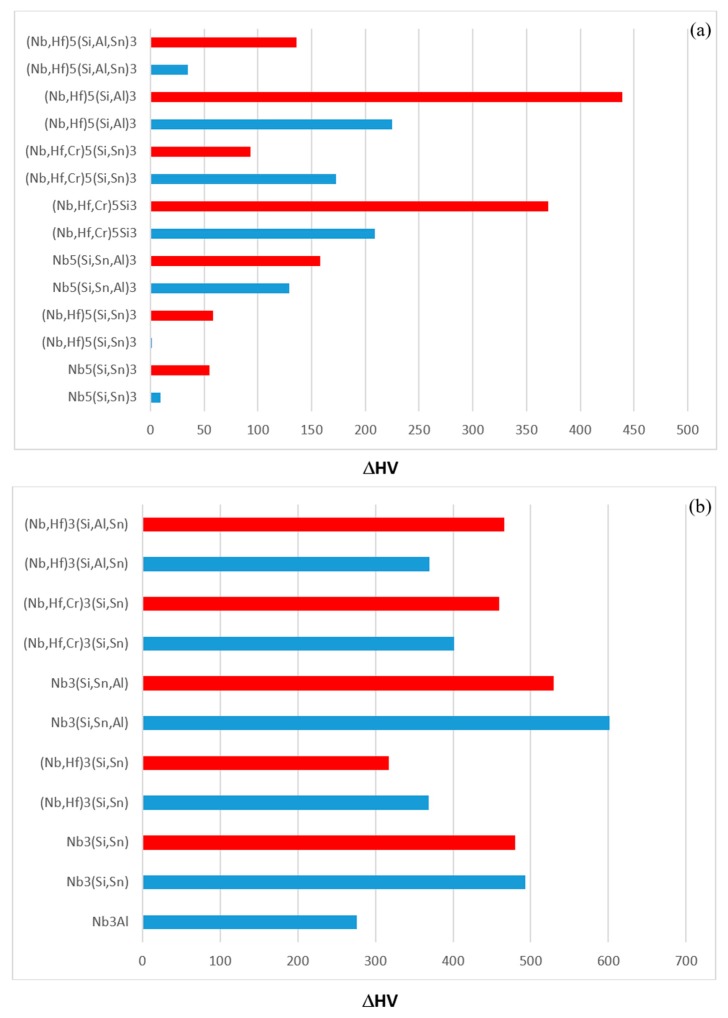
The hardness of alloyed Nb_5_Si_3_ (**a**) and Nb_3_Sn (**b**). Part (**a**) shows the reduction of the hardness of alloyed Nb_5_Si_3_ compared with the hardness of the unalloyed Nb_5_Si_3_. Part (**b**) shows the increase of the hardness of alloyed Nb_3_Sn compared with that of the unalloyed Nb_3_Sn. Blue and red colour respectively for the AC and HT condition.

The hardness values of the alloyed Nb_5_Si_3_ and Nb_3_Sn in the alloys EZ1, EZ7, EZ3 and EZ4 are compared with those of the unalloyed phases in Figure 14. The hardness of alloyed Nb_5_Si_3_ was reduced compared with the binary Nb_5_Si_3_ silicide. For example, Figure 14a shows that the hardness of (Nb,Cr,Hf)_5_Si_3_ was lower by 370 HV than that of the binary Nb_5_Si_3_ and that the hardness of (Nb,Hf)_5_(Si,Sn)_3_ was essentially the same as that of Nb_5_Si_3_. When Nb was substituted by Hf and Si by Al the reduction in the hardness of Nb_5_Si_3_ was the highest. The trends in the hardness data of Nb_5_Si_3_ are consistent with [4]. The hardness of alloyed Nb_3_Sn was increased compared with the binary Nb_3_Sn. For example, Figure 14b shows that the hardness of Nb_3_(Si,Sn,Al) and Nb_3_Al respectively was higher by 601 HV and 466 HV than that of the binary Nb_3_Sn. The trends in the hardness data of Nb_3_Sn are consistent with [5].

**Figure 15:**
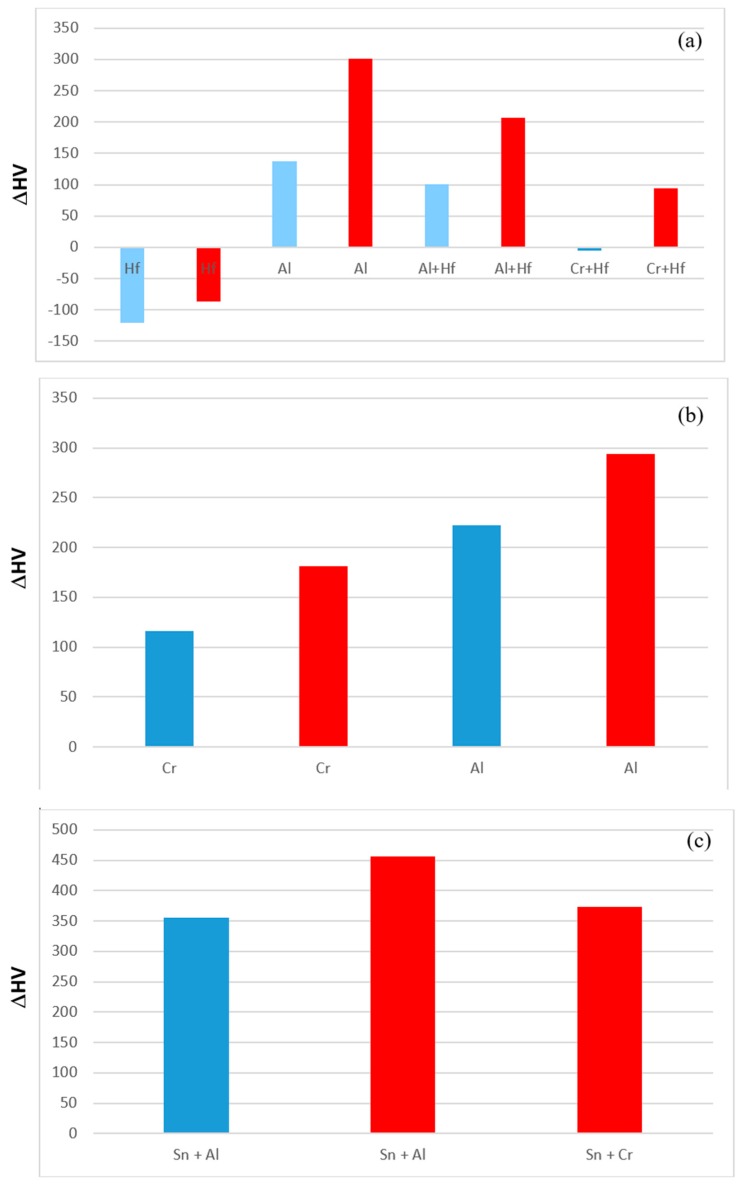
Effect of alloying addition on alloy hardness using as reference (**a**) the alloy Nb-18Si-5Sn (NV9 [14]), (**b**) the alloy EZ1 and (**c**) the alloys Nb-18Si-5Cr-5Hf (YG1 [17]) and Nb-18Si-5Al-5Hf (YG2 [17]). Blue and red colour respectively for the AC and HT condition.

The effect of alloying addition(s) on the hardness of the alloys is shown in Figure 15. It is possible to identify the effects of alloying elements individually and simultaneously using as reference the data for the alloys Nb-18Si-5Sn (NV9) (Figure 15a), EZ1 (Figure 15b) and YG1 and YG2 (Figure 15c). Aluminium on its own or together with Hf had a stronger effect than Cr (Figure 15b) and Cr + Hf (Figure 15a). Aluminium with Sn had a stronger effect than Cr with Sn.

The hardness of the alloys was calculated as described in [14] using HV = ∑ν_i_H_νi_ (law of mixtures) or HV^2^ = ∑(ν_i_H_νi_)^2^ (Pythagorean type addition rule) or 1/HV = ∑ν_i_/H_νi_ (an inverse type addition rule), where ν_i_ is the area fraction of a phase and H_νi_ is its hardness. The data is given in Table 4 together with the average measured hardness values from Table A1 in the Appendix A. For these calculations the hardness of 900 HV was used for the NbCr_2_ Laves phase [40,41,42]. The calculated hardness values that are close to the measured ones are given in bold numbers. Better agreement between experimental and calculated values is shown with the average of the Pythagorean and inverse type addition rules or with the average of the law of mixtures, Pythagorean and inverse type addition rules.

## 5. Oxidation

The addition of Sn in Ti containing Nb-silicide based alloys is known to suppress pest oxidation [9,11,43,44]. The volume fraction of the Nb_ss_ also is known to be critical for the oxidation of these alloys, with high volume fractions of the solid solution expected to have a strong detrimental effect. The Nb_5_Si_3_ is known to pest. All the alloys suffered pest oxidation at 800 °C. There was no Nb_ss_ in the Al containing alloys EZ7 and EZ4. The rapid and catastrophic pest oxidation of these two alloys was attributed to their intermetallic based microstructures. The pest oxidation of the alloy EZ3 that was “slightly better” compared with the other alloys was attributed to the presence of the C14 NbCr_2_ Laves phase in its microstructure.

## 6. Conclusions

We studied large (≈0.6 kg) arc melted buttons of the Nb-18Si-5Hf-5Sn (EZ1), Nb-18Si-5Al-5Sn (EZ7), Nb-18Si-5Cr-5Hf-5Sn (EZ3) and Nb-18Si-5Al-5Hf-5Sn (EZ4) alloys in the as-cast and heat treated conditions. We found that there was macrosegregation of Si (MACSi) in all the alloys. Also we found (i) that among the single element additions, Hf had the weakest and Sn the strongest effect on MACSi, and (ii) that the synergy of Cr and Hf had the strongest effect on MACSi. In all the alloys the βNb_5_Si_3_ was the primary phase and was present after the heat treatment(s), the Nb_3_Si silicide was suppressed and the A15-Nb_3_Sn intermetallic was stable. The Nb_ss_ was not stable in the alloys EZ7 and EZ4. Very Hf-rich Nb_5_Si_3_ was stable in the alloy EZ4 after prolonged heat treatments. Eutectics were observed in all the alloys and consisted on the Nb_ss_ and βNb_5_Si_3_, and βNb_5_Si_3_ and A15-Nb_3_Sn phases, respectively in the alloys EZ1 and EZ7 and most likely of the Nb_ss_, C14-NbCr_2_ and βNb_5_Si_3_, and Nb_ss_, βNb_5_Si_3_ and A15-Nb_3_Sn phases, respectively in the alloys EZ3 and EZ4. The addition of Al increased the vol% of the Nb_5_Si_3_ and A15-Nb_3_Sn phases, particularly after the heat treatment(s). The lattice parameter of Nb respectively increased and decreased with the addition of Hf, and Al or Cr and the latter element had the stronger negative effect. Pest oxidation was not suppressed in the Ti-free alloys of this study.

## Figures and Tables

**Table 1:** Phases in the cast and heat treated alloys EZ1, EZ3, EZ4 and EZ7. See the Tables S1–S4 in the Supplemental data for actual compositions.

Alloy	As Cast	Heat Treated			
		1500 °C			1200 °C
		Time (h)			
		100	200	300	100
EZ1	Nb_ss_, Hf rich Nb_ss_ Nb_3_Sn, Hf rich Nb_3_Sn α, β Nb_5_Si_3_, Hf rich Nb_5_Si_3_ (Nb_ss_ + Nb_5_Si_3_)_eut_ HfO_2_	Nb_ss_ Nb_3_Sn α, β Nb_5_Si_3_, Hf rich Nb_5_Si_3_ HfO_2_	Nb_ss_ Nb_3_Sn α, β Nb_5_Si_3_, Hf rich Nb_5_Si_3_ HfO_2_		
EZ7	Nb_3_Sn, Sn rich Nb_3_Sn α, β Nb_5_Si_3_ (Nb_3_Sn + Nb_5_Si_3_)_eut_	Nb_3_Sn α, β Nb_5_Si_3_			
EZ3	Nb_ss_ Nb_3_Sn α, β Nb_5_Si_3_ C14-NbCr_2_ (Nb_ss_ + NbCr_2_ + Nb_5_Si_3_)_eut_ HfO_2_				Nb_ss_ Nb_3_Sn α, β Nb_5_Si_3_, Hf rich Nb_5_Si_3_ C14 NbCr_2_ HfO_2_
EZ4	Nb_ss_ Nb_3_Sn α, β, γ Nb_5_Si_3_, Hf rich Nb_5_Si_3_ (Nb_ss_ + Nb_5_Si_3_)_eut_ HfO_2_	Nb_3_Sn α, β, γ Nb_5_Si_3_, Hf rich Nb_5_Si_3_ HfO_2_	Nb_ss_ Nb_3_Sn α, β, γ Nb_5_Si_3_, Hf rich Nb_5_Si_3_ Very Hf rich Nb_5_Si_3_ HfO_2_	Nb_3_Sn α, β, γ Nb_5_Si_3_, Hf rich Nb_5_Si_3_ Very Hf rich Nb_5_Si_3_ HfO_2_	

**Table 2:**
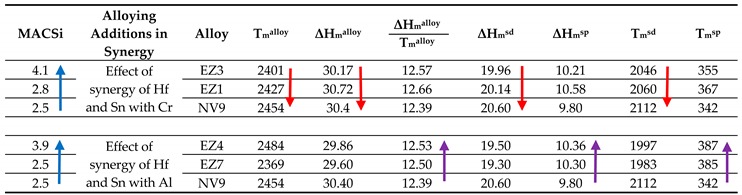
Effect of Al or Cr addition on the macrosegregation of Si in Nb-18Si based alloys with Hf and Sn and without Ti. The arrows indicate increase of corresponding parameter.

**Table 3:** Comparison of compositions (at.%) of Nb_ss_, Nb_3_Sn, Sn rich Nb_3_Sn, Nb_5_Si_3_ and Hf rich Nb_5_Si_3_ in the as-cast Nb silicide based alloys EZ1, EZ7, EZ3, EZ4 and NV9 (=Nb-18Si-5Sn [14]).

Phase	Solute Function	Alloy				
		EZ1	EZ3	EZ4	EZ7	NV9
Nb_ss_	Si/Sn	0.3	0.3			0.3
	Si/(Sn + Al)			0.21		
Nb_3_Sn	Si + Sn	17.2	18.2			17.8
	Si + Sn + Al			19.5	19.6	
Sn rich Nb_3_Sn	Si + Sn + Al				19.9	
Hf rich Nb_3_Sn	Si + Sn	18.1				
Nb_5_Si_3_	Si + Sn	38.4	38.6			36.2
	Si + Sn + Al			37.7	36.7	
Hf rich Nb_5_Si_3_	Si + Sn	38.4	38.8			
	Si + Sn + Al			38.3		
Eutectic with Nb_ss_ and Nb_5_Si_3_	Si + Sn	21.3				20.5
	Si + Sn + Al			21.7		

**Table 4:** Measured and calculated hardness values of the alloys.

Alloy and Condition	Hardness							
	Measured ^a^	Calculated ^b^						
		A	B	C	(A + B)/2	(B + C)/2	(A + C)/2	(A + B + C)/3
EZ1 AC	***693***	944	635	824	790	**730**	884	801
EZ1 HT1	***588***	702	412	605	557	509	654	**573**
EZ1 HT2	***592***	715	421	610	568	516	663	**582**
EZ7 AC	***952***	1143	812	1136	977	**974**	1139	1030
EZ7 HT	***977***	1093	779	1081	936	938	1087	**984**
EZ3 AC	***809***	948	626	888	787	757	918	**830**
EZ3 HT	***769***	975	602	915	788	**759**	945	830
EZ4 AC	***915***	1048	746	955	897	850	1002	**916**
EZ4 HT1	***882***	1104	808	1082	956	**945**	1068	998
EZ4 HT2	***887***	1051	745	1032	898	**889**	1042	943
EZ4 HT3	***879***	1040	738	1022	889	**880**	1031	933

^a^ see Table A1 in the Appendix A. ^b^ A—Law of mixtures, B—Pythagorean type additional rule, C—Inverse type addition rule (see text).

## Supplementary Materials

The following are available online at http://www.mdpi.com/1996-1944/11/12/2447/s1, Table S1: EPMA data (at.%) of the as-cast and heat treated EZ1 alloy, Table S2: EPMA data (at.%) of the as-cast and heat treated EZ7 alloy, Table S3: EPMA data (at.%) of the as-cast and heat treated alloy EZ3, Table S4: EPMA data (at.%) of the as-cast and heat treated alloy (EZ4). On the Nb-Si-Sn liquidus projection, Figure S1: Back scatter electron images of the alloy NV9 (Nb-18Si-5Sn) (a) and (b) as-cast, (c) heat treated 1500 °C for 100 h. For each part of the figure the analysis data (at.%) is given for the indicated analysis numbers. In (a) the contrast has been enhanced to show the Nb_ss_ and its different contrast from the A15-Nb_3_Sn phase.

## Appendix A

**Table A1:** Density, Vickers hardness (HV) of the alloys and phases and % areas of phases in their microstructures.

Alloy and Condition	Density (g/cm^3^)	Alloy Hardness (HV10)	% Areas of Phases in the Alloys			Microhardness of Phases		
			Nb_5_Si_3_	Nb_3_Sn	Nb_ss_	Nb_5_Si_3_	Nb_3_Sn	Nb_ss_
EZ1-AC	**8.35** ± 0.01 8.33–8.36	**693** ± 21 664–722	**42** ± 5	**28** ± 2	**28** ± 1	**1359** ± 68 1190–1616	**819** ± 25 712–919	**475** ± 20 411–538
EZ1-HT1	**8.18** 8.17–8.18	**588** ± 30 533–589	**19** ± 1	**23** ± 2		**1302** ± 61 1109–1481	**767** ± 37 710–800	–
EZ1- HT2	**8.32** ± 0.01 8.30–8.34	**592** ± 15 575–627	**21** ± 1	**22** ± 1		**1311** ± 54 1150–1469	**760** ± 43 704–814	–
EZ7-AC	**7.59** ± 0.01 7.58–7.61	**952** ± 7 919–988	**51** ± 2	**49** ± 2		**1231** ± 24 1105–1317	**1051** ± 24 998–1119	–
EZ7-HT	**7.75** ± 0.01 7.74–7.76	**977** ± 29 933–1026	**51** ± 3	**49** ± 3		**1202** ± 42 1098–1226	**979** ± 13 931–1002	–
EZ3-AC	**7.91** ± 0.01 7.90–7.93	**809** ± 12 743–846	**47** ± 2	**14** ± 1	**36** ± 2	**1187** ± 13 1063–1299	**851** ± 19 762–906	**677** ± 21 624–743
EZ3-HT	**8.19** ± 0.01 8.17–8.20	**769** ± 20 736–790	**40** ± 3.0	**35** ± 2		**1267** ± 51 1101–1441	**909** ± 13 879–927	–
EZ4-AC	**8.05** ± 0.01 8.03–8.07	**915** ± 18.9 880–939	**51.0** ± 4.4	**37.7** ± 2.4	**11.3** ± 2.1	**1325** ± 51 1194–1440	**820** ± 24 755–833	**559** ± 27 498–583
EZ4-HT1	**8.07** ± 0.01 8.05–8.08	**882** ± 25.7 842–925	**61.0** ± 4.4	**39.0** ± 3.4		**1224** ± 62 996–1306	**916** ± 63 825–1116	–
EZ4-HT2 *	**8.08** ± 0.01 8.06–8.09	**887** ± 14.5 873–916	**39.5** ± 3.8	**60.5** ± 5.0		**1230** ± 57 1001–1353	**934** ± 57 814–1089	–
EZ4-HT3 *	**8.11** ± 0.02 8.08–8.013	**879** ± 18.3 854–921	**39.5** ± 3.8	**60.5** ± 5.0		**1217** ± 43 1015–1320	**925** ± 48 854–1098	–

* The area fraction of the very Hf-rich Nb_5_Si_3_ phase (≈2.7%) was added to the total Nb_5_Si_3_ area.

**Table A2:** Lattice parameters of the Nb solid solution in the alloys NV9 * [14], EZ1, EZ3 and EZ4.

Alloy and Condition	Lattice Parameter (Å)
NV9-AC	3.125
NV9-HT (1500 °C/100 h)	3.127
EZ1-AC	3.299
EZ1-HT1 (1500 °C/100 h)	3.325
EZ1-HT2 (1500 °C/200 h)	3.310
EZ3-AC	3.285
EZ3-HT	3.298
EZ4 AC	3.298

* NV9 = Nb-18Si-5Sn.

**Table A3:** Type of Nb_5_Si_3_ in as-cast and heat treated small buttons, suction cast bars, large buttons and DS bars (see text) of Nb-silicide based alloys with/out addition of Ti.

Alloy	Code	As Cast				Heat Treated										Ref
						100 h				200 h	100 h				200 h	
						Temperature (°C)										
Nominal Composition (at.%)		Arc Melted			DS	1200	1400	1500	1500	1500	1300	1400	1500	1500	1500	
		SB	SC	LB	OFZ	SB			SC	SB	LB			OFZ	LB	
Nb-21.1Si-8.3Ti-5.4Mo-4W-0.7Hf	CM1	β	β	α	α			α	α				α	α		[19]
Nb-18Si-5Ge	ZF1	β				β, α				α						[45]
Nb-18Si-10Ge	ZF2	β				β, α				α						[45]
Nb-14Si-3Sn	ZX1	β						α								[46]
Nb-12.5Si-7.5Sn	ZX2	β						α								[46]
Nb-19.1Si-1.5In		β, α														[47]
Nb-20.2Si-2.7Ga		β														[48]
Nb-20Si-xMo (x = 2, 4, 6)		β														[49]
Nb-18Si-5Cr-5Ge	ZF7	β					β, α									[50]
Nb-18Si-5Cr-5Hf	YG1			β, α									β, α			[17]
Nb-18Si-5Al-5Ge	ZF8	β					β, α									[51]
Nb-18Si-5Al-5Hf	YG2			β, α									β, α			[17]
Nb-17Si-10Mo-3Al				β	β											[52]
Nb-20Si-5Hf-5W	YG5			β, α									β, α			[53]
Nb-20Si-5Hf-5Mo-3W	YG8			β, α									β, α			[53]
Nb-18Si-5Al-5Cr-5Mo	JG1			β									α		α	[54]
Nb-24Ti-18Si-5Sn	NV6			β, α		α										[14]
Nb-24Ti-18Si-5Al	KZ7			β									α			[38]
Nb-24Ti-18Si-4Al-8Cr	KZ2			β								β, α	β, α			[31]
Nb-24Ti-18Si-5Al-5Cr	KZ5			β									β, α		β, α	[31]
Nb-24Ti-18Si-5Al-5Ge	ZF5	β					β, α									[51]
Nb-24Ti-18Si-5Cr-5Ge	ZF4	β					β, α									[50]
Nb-24Ti-18Si-5Al-5Cr-6Ta	KZ6			β									β, α		β, α	[38]
Nb-24Ti-18Si-4Al-8Cr-6Ta	KZ8			β								β, α	β, α			[38]
Nb-21Ti-16Si-3Al-7Cr-2Hf				β, α	β, α											[55]
Nb-22Ti-14Si-2Al-4Cr-2Hf					α *									α *		[56]
Nb-24Ti-18Si-5Al-5Cr-5Mo	JG2			β									β, α			[54]
Nb-24Ti-18Si-5Al-5Cr-2Mo	JG3			β									β, α			[54]
Nb-24Ti-18Si-5Al-5Cr-5Hf-2Mo	JG4			β									β, α			[57]
Nb-24Ti-18Si-5Al-5Cr-5Hf-2Mo-5Sn	JG6			β							β, α					[57]
Nb-14Si-24Ti-10Cr-2Al-2Hf-0.1Y					α *									α *^,+^		[58]

SB = small button, SC = suction cast bar, LB = large button, OFZ = optical floating zone melting. β = βNb_5_Si_3_, α = αNb_5_Si_3_. * Liquid-metal-cooled directional solidification (LMC) ^+^ HT = 1450 °C/10 h.
