# Supplementary material for: A Study of the Effects of Hf and Sn on the Microstructure, Hardness and Oxidation of Nb-18Si Silicide Based Alloys without Ti Addition
Source: Materials (Basel). 2018 Dec 3;11(12):2447. doi: 10.3390/ma11122447 (PMC6317196; doi:10.3390/ma11122447)
Supplement: Supplementary file 1 [file materials-11-02447-s001.pdf]

Supplementary

A Study of the Effects of Hf and Sn on the Microstructure, Hardness and Oxidation of Nb – 18Si Silicide Based Alloys without Ti Addition

Eleftherios Zacharis, Claire Utton and Panos Tsakiropoulos *

Department of Materials Science and Engineering, Sir Robert Hadfield Building, University of Sheffield, Mappin Street, Sheffield S1 3JD, UK; lefteris.zacharis@alfagro.gr (E.Z.); c.utton@sheffield.ac.uk (C.U.)

* Correspondence: p.tsakiropoulos@sheffield.ac.uk

Table S1 EPMA data (at.%) of the as-cast and heat treated EZ1 alloy.

Condition and phase	Nb	Si	Hf	Sn
**As cast EZ1 (EZ1-AC)**				
Average composition	69.5-71.9<br>**70.0±0.3**	18.9-21.7<br>**20.5±0.2**	5.1-5.7<br>**5.4±0.1**	3.9-4.3<br>**4.1±0.1**
Nb_{ss}	85.1-90.1<br>**87.5±0.4**	1.3-4.0<br>**2.1±0.3**	3.3-4.8<br>**4.3±0.2**	3.6-7.7<br>**6.1±0.3**
Hf rich Nb_{ss}	79.1-87.6<br>**82.5±0.5**	1.3-2.7<br>**1.9±0.2**	5.5-9.0<br>**7.5±0.2**	4.9-9.8<br>**8.1±0.3**
Eutectic with Nb_{ss} and Nb_5Si_3	70.4-76.0<br>**73.7±0.6**	15.8-20.1<br>**17.7±0.4**	4.3-6.5<br>**5.0±0.3**	2.9-4.3<br>**3.6±0.2**
Nb_3Sn	79.2-81.3<br>**80.4±0.2**	6.5-8.0<br>**7.2±0.2**	2.0-3.2<br>**2.4±0.2**	9.4-11.0<br>**10.0±0.3**
Hf rich Nb_3Sn	72.4-77.6<br>**75.5±0.3**	3.8-6.5<br>**5.4±0.2**	4.3-9.1<br>**6.3±0.2**	11.3-14.8<br>**12.7±0.2**
Nb_5Si_3	53.1-57.0<br>**56.1±0.4**	34.6-37.4<br>**36.9±0.3**	4.8-7.9<br>**5.5±0.2**	1.1-2.2<br>**1.5±0.2**
Hf rich Nb_5Si_3	47.8-54.8<br>**52.1±0.3**	30.3-37.8<br>**36.1±0.3**	8.0-15.0<br>**9.5±0.6**	1.3-4.8<br>**2.3±0.3**
**Heat-treated 1 EZ1 1500 °C/100h (EZ-HT1)**				
Bulk	66.6-70.5<br>**68.8±0.3**	19.8-24.0<br>**21.4±0.6**	5.4-5.8<br>**5.6±0.1**	3.9-4.3<br>**4.2±0.1**
Nb_{ss}	87.9-94.1<br>**92.6±0.2**	1.3-2.1<br>**1.6±0.2**	2.4-3.8<br>**2.8±0.3**	3.0-3.7<br>**3.4±0.1**
Nb_3Sn	78.1-80.1<br>**79.1±0.2**	4.0-6.3<br>**4.5±0.4**	2.5-3.2<br>**2.9±0.2**	12.4-14.1<br>**13.5±0.3**
Prior eutectic areas	68.8-70.4<br>**69.6±0.7**	22.1-22.2<br>**22.1±0.0**	6.1-6.3<br>**6.2±0.0**	2.4-2.8<br>**2.1±0.1**
Nb_5Si_3	55.4-57.0<br>**56.5±0.2**	36.5-37.8<br>**37.1±0.3**	4.7-5.8<br>**5.0±0.2**	1.0-1.9<br>**1.4±0.2**
Hf rich Nb_5Si_3	50.9-59.3<br>**54.1±0.5**	29.2-39.8<br>**36.0±0.4**	6.4-9.8<br>**7.8±0.3**	1.3-5.0<br>**2.1±0.3**
**Heat-treated 2 EZ1 1500 °C/200h (EZ1-HT2)**				
Bulk	69.2-71.5<br>**70.2±0.5**	18.9-21.6<br>**20.4±0.6**	5.2-5.5<br>**5.4±0.1**	3.8-4.4<br>**4.1±0.1**
Nb_{ss}	92.0-94.1<br>**93.4±0.6**	0.0-2.0<br>**0.7±0.4**	2.4-2.8<br>**2.6±0.1**	3.1-3.6<br>**3.3±0.1**
Nb_3Sn	75.3-80.2<br>**79.0±0.9**	3.7-9.3<br>**5.0±0.8**	2.6-3.9<br>**2.9±0.3**	11.5-13.9<br>**13.1±0.3**
Prior eutectic areas	70.1-73.9<br>**72.3±0.6**	17.9-20.5<br>**19.5±0.7**	5.4-6.0<br>**5.8±0.2**	2.2-2.5<br>**2.4±0.1**

Nb_5Si_3	57.4-58.2 **57.8±0.2**	35.7-36.3 **36.0±0.2**	4.7-4.8 **4.8±0.0**	1.3-1.6 **1.4±0.1**
Hf rich Nb_5Si_3	50.3-57.1 **53.9±0.7**	36.3-37.1 **36.6±0.2**	5.6-10.5 **7.9±1.1**	1.1-2.2 **1.6±0.2**

Table S2 EPMA data (at.%) of the as-cast and heat treated EZ7 alloy

**Condition and phase**	**Nb**	**Si**	**Al**	**Sn**
**As cast EZ7 (EZ7-AC)**				
Average composition	71.5-73.0 **72.1±0.4**	17.6-20.1 **18.9±0.55**	4.9-5.4 **5.0±0.1**	3.5-4.5 **4.0±0.2**
Nb_3Sn	77.2-81.2 **80.4±0.7**	6.9-11.9 **7.7±0.8**	6.2-7.0 **6.5±0.2**	4.6-6.1 **5.4±0.4**
Sn rich Nb_3Sn	79.4-80.7 **80.1±0.3**	6.4-8.5 **7.1±0.4**	5.2-6.5 **5.8±0.4**	6.1-8.4 **7.0±0.6**
Nb_5Si_3	63.0-63.7 **63.3±0.2**	30.7-33.2 **32.0±0.6**	2.5-4.3 **3.2±0.4**	1.2-2.1 **1.5±0.3**
Nb_3Sn - Nb_5Si_3 eutectic	74.5-76.5 **75.7±0.6**	12.4-14.7 **13.4±0.6**	5.0-5.9 **5.4±0.2**	4.7-6.5 **5.5±0.5**
**Heat-treated EZ7 1500 ºC/100h (EZ7-HT)**				
Bulk	72.0-74.4 **73.0±0.8**	16.0-19.3 **18.1±1.1**	4.8-5.2 **5.0±0.2**	3.4-4.3 **3.9±0.3**
Nb_3Sn	81.5-82.6 **82.0±0.3**	4.9-6.3 **5.3±0.4**	6.0-6.7 **6.3±0.2**	5.0-7.2 **6.4±0.5**
Nb_5Si_3	64.4-64.8 **64.6±0.1**	30.6-31.7 **31.2±0.3**	2.6-3.0 **2.8±0.1**	1.1-1.5 **1.3±0.1**

Table S3 EPMA data (at.%) of the as-cast and heat treated alloy EZ3.

**Condition and phase**	**Nb**	**Si**	**Cr**	**Hf**	**Sn**
**As cast EZ3 (EZ3-AC)**					
Average composition	65.0-67.9 **66.2±0.25**	17.4-21.5 **19.7±0.35**	3.8-5.8 **4.5±0.2**	4.9-5.4 **5.3±0.1**	3.7-4.9 **4.5±0.25**
Nb_{ss}	77.2-83.8 **81.3±0.5**	1.0-3.3 **1.7±0.3**	5.1-11 **8.1±0.7**	2.1-3.9 **3.6±0.3**	4.4-7.3 **5.2±0.4**
Nb_3Sn	68.5-78.4 **76.1±0.4**	4.2-7.4 **6.4±0.3**	1.6-6.0 **3.3±0.6**	1.8-6.4 **2.4±0.6**	10.4-14.8 **11.8±0.6**
Nb_5Si_3	54.6-57.1 **56.0±0.6**	36.2-37.7 **37.0±0.3**	0.0-1.1 **0.1±0.1**	4.5-6.3 **5.3±0.3**	1.3-2.1 **1.6±0.3**
Hf rich Nb_5Si_3	51.6-55.3 **53.0±0.7**	33.2-38.0 **37.2±0.8**	0.0-2.0 **0.6±0.6**	6.1-8.3 **7.6±0.4**	1.1-3.0 **1.6±0.5**
Eutectic with Nb_{ss}, Nb_5Si_3 and C14 $NbCr_2$ Laves phase	31.9-53.6 **44.4±5.4**	7.2-13.4 **9.6±1.4**	30.3-49.5 **37.9±4.6**	4.7-10.4 **6.0±2.1**	0.0-3.1 **2.1±1.0**
**Heat-treated - EZ3 1200 ºC/100 h (EZ3-HT)**					
Bulk	63.6-66.6 **65.0±0.6**	18.4-21.8 **20.3±0.3**	4.3-6.6 **5.1±0.4**	5.2-6.5 **5.5±0.3**	3.7-5.2 **4.2±0.5**
Nb_{ss}	87.3-90.5 **89.0±0.6**	0.0-1.9 **0.7±0.4**	4.9-7.0 **5.7±0.4**	1.9-3.4 **2.4±0.4**	1.9-2.5 **2.2±0.3**
Nb_3Sn	74.5-78.7 **77.5±0.5**	3.7-6.8 **5.5±0.7**	2.3-6.3 **3.2±0.7**	1.8-3.0 **2.3±0.5**	10.4-12.8 **11.5±0.4**
Nb_5Si_3	54.8-57.0 **56.3±0.5**	36.5-37.5 **36.9±0.4**	0.0-0.7 **0.3±0.3**	4.6-6.1 **5.1±0.5**	1.1-1.7 **1.4±0.1**
Hf rich Nb_5Si_3	51.7-55.8 **53.4±0.9**	32.6-37.6 **36.6±0.8**	0.5-2.4 **0.9±0.4**	6.4-8.7 **7.6±0.6**	1.1-1.8 **1.5±0.2**
C14 $NbCr_2$ Laves phase	30.1-37.2	8.9-9.8	46.1-51.8	4.7-6.9	0.4-2.4

	**35.1±0.5**	**9.3±0.3**	**48.6±0.5**	**5.5±0.3**	**1.5±0.3**
Prior eutectic areas	42.7-46.0<br>**44.4±0.5**	8.5-8.8<br>**8.6±0.1**	37.6-43.0<br>**40.2±0.3**	4.5-5.1<br>**4.8±0.2**	0.6-4.6<br>**2.2±0.6**

Table S4 EPMA data (at.%) of the as-cast and heat treated alloy (EZ4).

Condition and phase	Nb	Si	Al	Hf	Sn
**As Cast EZ4 (EZ4-AC)**					
Average composition	65.1-70.0<br>**67.6±0.5**	17.4-21.3<br>**19.4±0.5**	4.0-5.2<br>**4.4±0.2**	5.0-5.6<br>**5.3±0.1**	2.9-4.5<br>**3.3±0.3**
Nb_{ss}	81.3-84.4<br>**82.9±0.6**	1.5-2.4<br>**1.9±0.2**	5.5-6.8<br>**6.2±0.3**	4.8-7.4<br>**6.2±0.3**	2.2-3.1<br>**2.8±0.2**
Nb_3Sn	75.6-79.0<br>**77.6±0.6**	4.8-9.3<br>**6.6±0.6**	4.4-7.3<br>**5.5±0.5**	2.0-4.1<br>**2.9±0.5**	5.4-8.8<br>**7.4±0.4**
Nb_5Si_3	57.6-59.0<br>**58.6±0.4**	32.4-33.9<br>**33.1±0.3**	2.6-4.0<br>**3.2±0.3**	3.5-4.0<br>**3.7±0.1**	1.0-1.8<br>**1.4±0.2**
Hf rich Nb_5Si_3	47.6-56.9<br>**53.7±1.3**	30.2-36.1<br>**33.3±1.2**	1.9-6.2<br>**3.8±0.9**	5.4-14.7<br>**8.0±1.1**	0.6-1.7<br>**1.2±0.2**
Eutectic with Nb_{ss} and Nb_5Si_3	68.6-71.0<br>**69.7±0.2**	14.5-15.2<br>**14.9±0.1**	4.3-4.7<br>**4.5±0.1**	8.0-9.1<br>**8.6±0.3**	2.3-2.4<br>**2.3±0.0**
**Heat-treated 1 EZ4 - 1500 ºC/100 h = EZ4-HT1**					
Bulk	66.5-67.7<br>**67.1±0.3**	19.0-20.3<br>**19.8±0.3**	4.2-4.8<br>**4.4±0.1**	5.1-5.7<br>**5.4±0.1**	3.1-3.4<br>**3.2±0.1**
Nb_3Sn	78.6-81.8<br>**79.7±0.4**	3.9-5.4<br>**4.8±0.5**	6.2-7.2<br>**6.7±0.2**	0.3-2.7<br>**1.9±0.7**	6.4-7.4<br>**6.9±0.3**
Nb_5Si_3	56.5-59.5<br>**58.3±0.6**	32.9-34.9<br>**33.9±0.3**	2.4-3.1<br>**2.8±0.1**	3.6-4.9<br>**3.9±0.3**	0.6-1.4<br>**1.1±0.2**
Hf-rich Nb_5Si_3	50.8-55.1<br>**52.8±0.6**	33.7-36.5<br>**35.6±0.6**	2.2-3.0<br>**2.5±0.2**	6.6-9.8<br>**8.2±0.5**	0.6-1.1<br>**0.9±0.1**
**Heat-treated 2 EZ4 - 1500 ºC/200 h = EZ4-HT2**					
Bulk	67.7-69.4<br>**68.5±0.5**	18.3-19.6<br>**18.9±0.3**	3.9-4.4<br>**4.2±0.1**	4.8-5.6<br>**5.2±0.2**	2.9-3.7<br>**3.3±0.3**
Nb_{ss}*	91.9-92.8<br>**92.4**	0.7-0.8<br>**0.7**	2.7-3.1<br>**2.9**	2.3-2.8<br>**2.5**	1.4-1.5<br>**1.5**
Nb_3Sn	79.4-80.3<br>**79.8±0.3**	3.9-5.0<br>**4.3±0.3**	6.4-7.2<br>**6.8±0.2**	2.1-2.5<br>**2.3±0.1**	6.4-7.6<br>**6.9±0.2**
Nb_5Si_3	56.1-59.3<br>**57.9±0.9**	32.0-34.6<br>**33.6±0.8**	2.1-3.6<br>**2.7±0.5**	3.7-5.9<br>**4.7±0.8**	1.0-1.4<br>**1.1±0.1**
Hf-rich Nb_5Si_3	52.5-56.2<br>**54.1±0.7**	31.5-35.0<br>**34.1±0.7**	2.4-3.1<br>**2.7±0.2**	6.4-9.6<br>**8.1±0.9**	0.6-1.2<br>**0.9±0.2**
Very Hf-rich Nb_5Si_3	38.0-46.7<br>**41.2±2.3**	31.1-34.7<br>**33.7±0.8**	5.0-5.7<br>**5.4±0.2**	16.4-21.4<br>**19.5±1.5**	0.0-0.5<br>**0.2±0.1**
**Heat-treated 3 EZ4 - 1500 ºC/300 h = EZ4-HT3**					
Bulk	66.9-68.6<br>**68.0±0.4**	18.1-19.2<br>**18.6±0.4**	3.8-5.0<br>**4.3±0.4**	4.9-5.9<br>**5.4±0.3**	3.5-4.0<br>**3.7±0.1**
Nb_3Sn	79.4-79.9<br>**79.6±0.1**	4.2-4.4<br>**4.3±0.0**	6.1-7.1<br>**6.7±0.3**	2.5-2.5<br>**2.5±0.0**	6.3-6.9<br>**6.7±0.2**
Nb_5Si_3	57.2-57.9<br>**57.6±0.2**	33.1-33.6<br>**33.4±0.1**	3.0-3.2<br>**3.1±0.0**	3.8-5.0<br>**4.4±0.3**	1.1-1.8<br>**1.5±0.3**
Hf-rich Nb_5Si_3	53.5-55.6<br>**54.4±0.5**	33.4-34.2<br>**33.7±0.2**	2.0-2.7<br>**2.4±0.2**	7.4-9.0<br>**8.4±0.3**	0.8-1.4<br>**1.1±0.2**
Very Hf-rich Nb_5Si_3	40.2-42.1<br>**41.3±0.5**	32.1-34.4<br>**33.2±0.4**	4.7-6.4<br>**5.4±0.4**	18.3-19.9<br>**19.2±0.4**	0.0-0.5<br>**0.2±0.1**

*Only two analyses were possible owing to the size of this phase.

On the Nb-Si-Sn liquidus projection

The liquidus projection for the Nb-Si-Sn system has areas for βNb_5Si_3 and αNb_5Si_3 [1]. At 1974 ^oC it gives the invariant reaction L1 + $\beta Nb_5Si_3 \rightarrow$ L2 + αNb_5Si_3 for the transformation of βNb_5Si_3 to αNb_5Si_3, where L1 and L2 result from a miscibility gap in the Si-Sn binary, and at 1874 ^oC gives the invariant reaction L $\rightarrow$ (Nb) + A15 + αNb_5Si_3 [1]. Unfortunately, the paper by Sun et al [1] is inconsistent with the experimental work reported in [2] for the alloy Nb-18Si-5Sn (alloy NV9), the results of which were used by Sun et al. According to Sun et al (i) "the divorced eutectic of Nb_5Si_3 + A15_Nb_3Sn" was reported in the ref.[7] in their paper and (ii) "the fine eutectic was roughly identified as (Nb) + Nb_5Si_3 binary eutectic in samples from top and centre of the ingot, while it was identified as (Nb) + Nb_5Si_3 + A15_Nb_3Sn in sample from the bottom of the ingot [7]" (ref.[7] in Sun et al is the paper by Vellios and Tsakiropoulos, which is the reference [2]). Vellios and Tsakiropoulos [2] did not report a divorced eutectic of Nb_5Si_3 + A15-Nb_3Sn and did not identify the eutectic in the bottom of the ingot as (Nb) + Nb_5Si_3 + A15_Nb_3Sn. Instead, they reported that only the Nb_{ss} + Nb_5Si_3 eutectic was observed in all parts of the as-cast alloy. Figure S1 shows the microstructure of the alloy NV9 in the as-cast condition (Figures S1a and S1b), together with analysis data for the indicated phases and areas. Figures S1a and S1b clearly show that the lamellar microstructure consisted of the Nb_{ss} and Nb_5Si_3 phases. Figure S1 is given to highlight how difficult can be the identification of Nb_{ss} and Nb_3Sn in the microstructures of Nb-silicide based alloys. This difficulty increases further when Hf is present in the alloy owing to the partitioning of the element between the phases.

Referring to ref.[7] in their paper, Sun et al also stated "since a small fraction of Nb_3Sn was found between Nb_5Si_3 and (Nb) in the 1200 ^oC, 1500 ^oC and 1600 ^oC heat treated samples, it seems from the non-equilibrium as-cast microstructure, this evidence may lend certain support to (Nb) + Nb_5Si_3 + A15_Nb_3Sn ternary eutectic in the as-cast alloy, and it is more reasonable to identify the fine eutectic as (Nb) + Nb_5Si_3 + A15_Nb_3Sn ternary eutectic in the cast microstructures" [1]. Figure S1c shows the microstructure of the alloy NV9 after the heat treatment at 1500 ^oC for 100 h, together with analysis data for the indicated phases. Figure S1c clearly shows that after the heat treatment the prior lamellar microstructure areas still consisted of the Nb_{ss} and Nb_5Si_3 phases.

The XRD data in [2] for the as-cast alloy NV9 had only one peak that corresponded only to αNb_5Si_3, 6 peaks that corresponded to other phases and αNb_5Si_3, and 4 peaks that corresponded to βNb_5Si_3 and other phases. Peaks were shared between the βNb_5Si_3 and other phases in the XRD data for the heat treated alloy NV9 at 1200, 1500 and 1600 ^oC, and one peak corresponded only to βNb_5Si_3 in the diffractograms for the 1200 and 1600 ^oC heat treatments. Sun et al [1] stated "for the as-cast and heat treated NV9 alloy, Nb_5Si_3 including the structures of both αNb_5Si_3 and βNb_5Si_3 were indicated by XRD results in Vellios et al.'s work, but no particular peaks of βNb_5Si_3 were found when we carefully checked the XRD results, so the identification of βNb_5Si_3 was not considered credible. However, the particular peaks of αNb_5Si_3 confirmed its presence, and the existence of αNb_5Si_3 is more reasonable to coincide with Waterstrat et al.'s work and its stability in the Nb-Si binary, especially for the heat treated samples, so the identification of αNb_5Si_3 is accepted for this modelling work" (the reference to Waterstrat et al is for the partial isothermal section of the Nb-Si-Sn system at 1600 ^oC which is given in ref. [6] in [1]. Waterstrat and Muller did not give a liquidus projection for Nb-Si-Sn in their paper (ref.6] in [1]).

References

1. Zhiping Sun, Xiping Guo, Chuan Zhang, Thermodynamic modelling of the Nb-rich corner in the Nb-Si-Sn system, CALPHAD: Computer coupling of phase diagrams and thermochemistry 36 (2102) 82-88

2. N Vellios, P Tsakiropoulos, The role of Sn and Ti additions in the microstructure of Nb-18Si based alloys, Intermetallics 15 (2007) 1518-1528

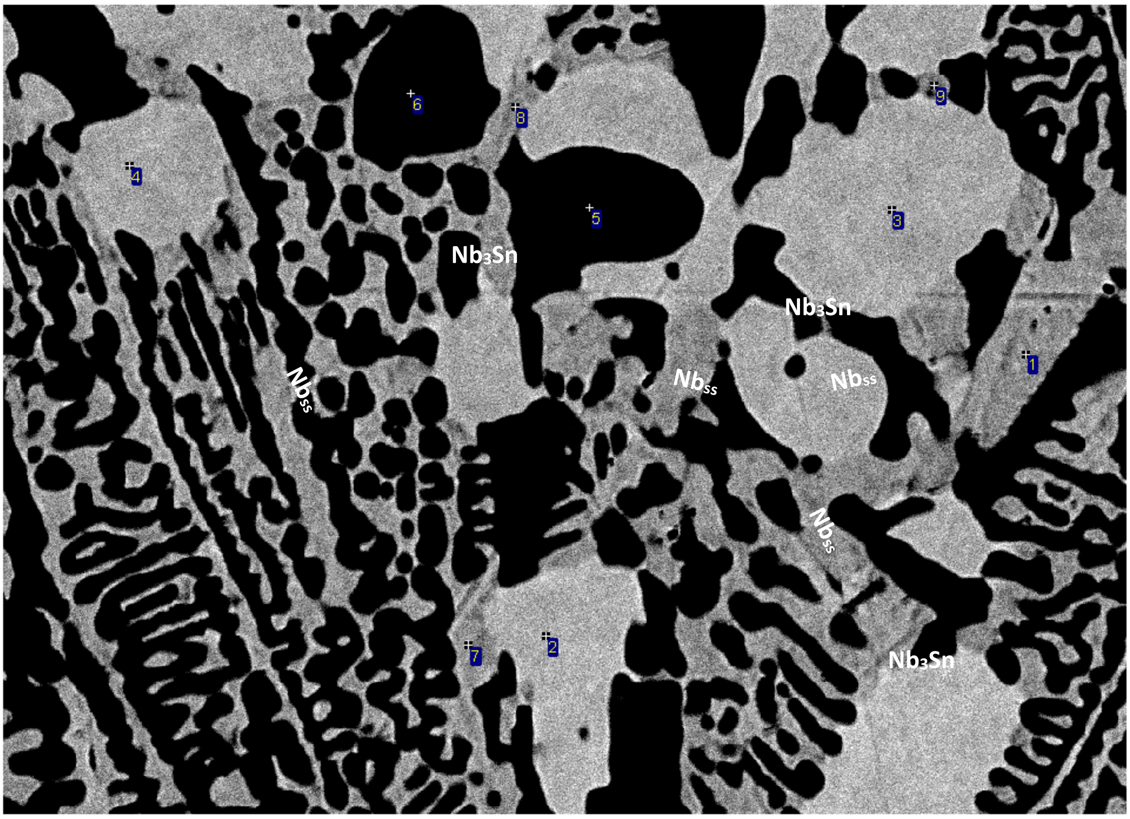

30μm Electron Image 1

Analysis number	Si	Nb	Sn	phase
1	1.35	94.44	4.21	Nb_{ss}
2	8.57	82.54	8.89	A15-Nb_3Sn
3	8.04	82.78	9.18	A15-Nb_3Sn
4	8.18	82.69	9.13	A15-Nb_3Sn
5	35.11	63.77	1.11	Nb_5Si_3
6	34.87	63.79	1.34	Nb_5Si_3
7	1.04	94.74	4.22	Nb_{ss}
8	1.19	94.77	4.04	Nb_{ss}
9	1.15	94.90	3.95	Nb_{ss}

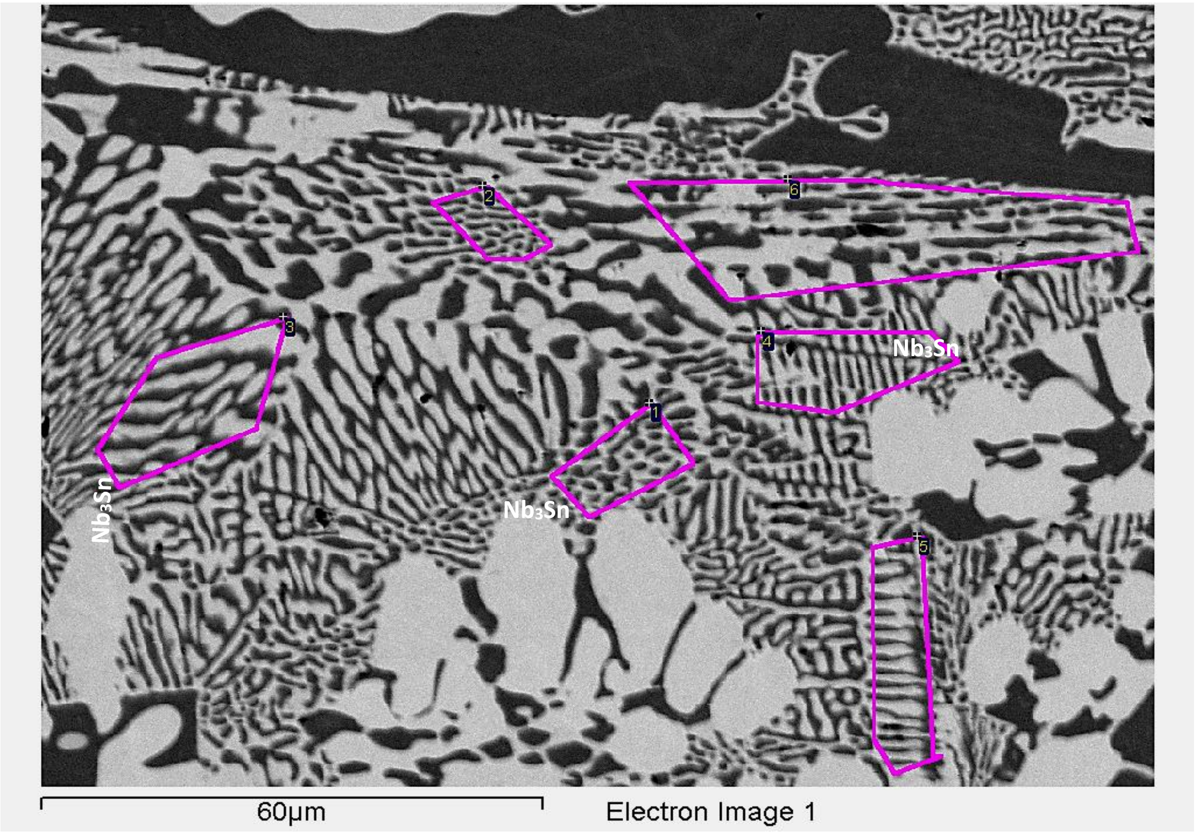

Area analysis number	Si	Nb	Sn
1	18.28	79.00	2.72
2	18.62	78.60	2.78
3	18.40	78.68	2.92
4	17.12	80.09	2.79
5	17.02	80.14	2.84
6	17.87	79.16	2.97
average	17.88	79.28	2.84

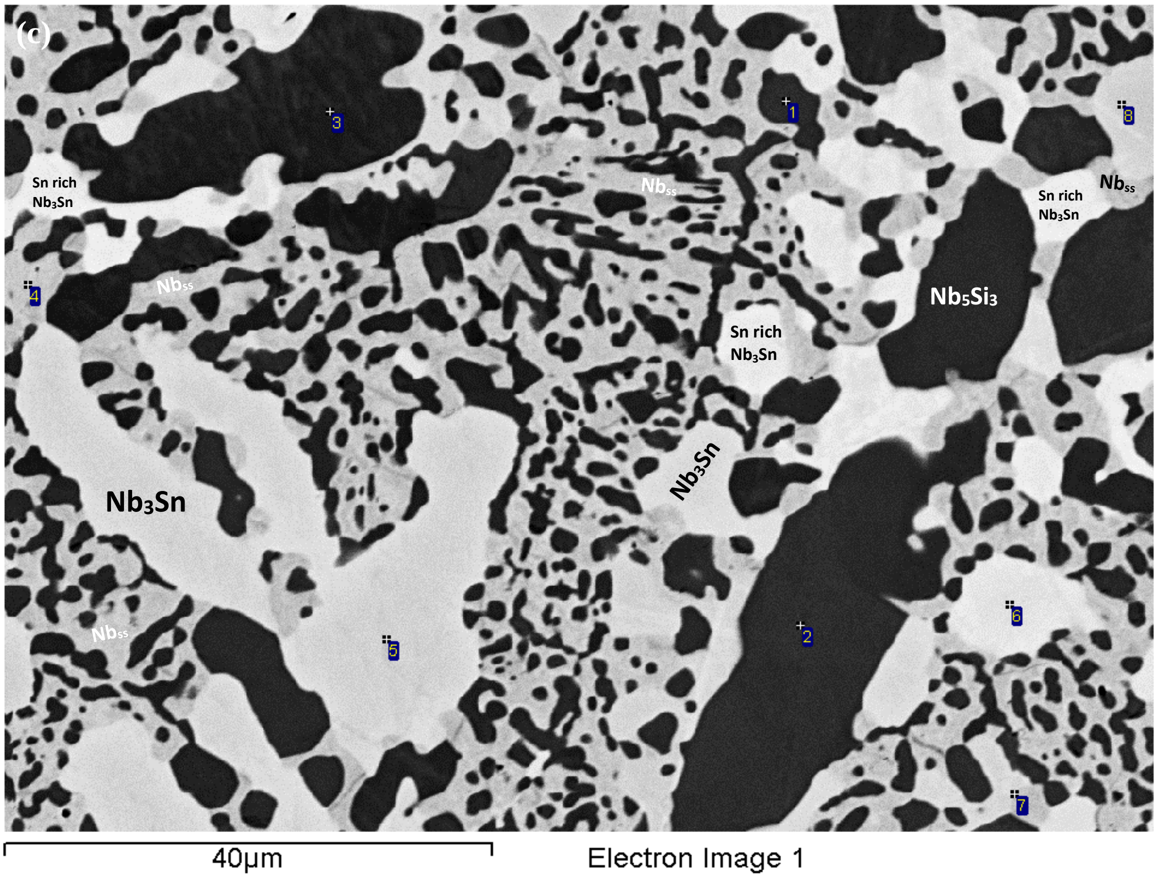

Analysis number	Si	Nb	Sn	Phase
1	35.74	63.59	0.67	Nb_5Si_3
2	35.03	63.76	1.21	Nb_5Si_3
3	35.32	63.29	1.39	Nb_5Si_3
4	1.29	95.84	2.87	Nb_{ss}
5	7.66	83.32	9.02	A15-Nb_3Sn
6	7.70	83.17	9.13	A15-Nb_3Sn
7	2.69	90.10	7.21	Nb_{ss}
8	7.60	82.67	9.73	A15-Nb_3Sn

Figure S1: Back scatter electron images of the alloy NV9 (Nb-18Si-5Sn) (a) and (b) as-cast, (c) heat treated 1500 ^{o}C for 100 h. For each part of the figure the analysis data (at.%) is given for the indicated analysis numbers. In (a) the contrast has been enhanced to show the Nb_{ss} and its different contrast from the A15-Nb_3Sn phase.
